# CystiHuman: A model of human neurocysticercosis

**DOI:** 10.1371/journal.pcbi.1010118

**Published:** 2022-05-19

**Authors:** Gabrielle Bonnet, Francesco Pizzitutti, Eloy A. Gonzales-Gustavson, Sarah Gabriël, William K. Pan, Hector H. Garcia, Javier A. Bustos, Percy Vilchez, Seth E. O’Neal, for the Cysticercosis Working Group in Peru

**Affiliations:** 1 Independent Consultant for the School of Public Health, Oregon Health & Science University, Portland, Oregon, United States of America; 2 Tropical and Highlands Veterinary Research Institute, University of San Marcos, Lima, Peru; 3 Department of Veterinary Public Health and Food Safety, Ghent University, Gent, Belgium; 4 Nicholas School of Environment and Duke Global Health Institute, Duke University, Durham, North Carolina, United States of America; 5 Center for Global Health Tumbes, Universidad Peruana Cayetano Heredia, Lima, Peru; 6 Cysticercosis Unit, National Institute of Neurological Sciences, Lima, Peru; 7 School of Public Health, Oregon Health & Science University and Portland State University, Portland, Oregon, United States of America; University of Notre Dame, UNITED STATES

## Abstract

**Introduction:**

The *Taenia solium* tapeworm is responsible for cysticercosis, a neglected tropical disease presenting as larvae in the body of a host following taenia egg ingestion. Neurocysticercosis (NCC), the name of the disease when it affects the human central nervous system, is a major cause of epilepsy in developing countries, and can also cause intracranial hypertension, hydrocephalus and death. Simulation models can help identify the most cost-effective interventions before their implementation. Modelling NCC should enable the comparison of a broad range of interventions, from treatment of human taeniasis (presence of an adult taenia worm in the human intestine) to NCC mitigation. It also allows a focus on the actual impact of the disease, rather than using proxies as is the case for other models.

**Methods:**

This agent-based model is the first model that simulates human NCC and associated pathologies. It uses the output of another model, CystiAgent, which simulates the evolution of pig cysticercosis and human taeniasis, adding human and cyst agents, including a model of cyst location and stage, human symptoms, and treatment. CystiHuman also accounts for delays in the appearance of NCC-related symptoms. It comprises three modules detailing cyst development, seizure probability and timing, and intracranial hypertension/hydrocephalus, respectively. It has been implemented in Java MASON and calibrated in three endemic villages in Peru, then applied to another village (Rica Playa) to compare simulation results with field data in that village.

**Results and discussion:**

Despite limitations in available field data, parameter values found through calibration are plausible and simulated outcomes in Rica Playa are close to actual values for NCC prevalence and the way it increases with age and cases with single lesions. Initial simulations further suggest that short-term interventions followed by a rapid increase in taeniasis prevalence back to original levels may have limited impacts on NCC prevalence.

## Author summary

This paper presents CystiHuman, the first model of human neurocysticercosis, a parasitic disease that is responsible for a large proportion of epilepsy cases in developing countries and also contributes to intracranial hypertension, hydrocephalus and death. Existing modelling efforts have focused so far on parasite transmission i.e., how the parasite responsible for neurocysticercosis, *Taenia solium*, persists through interactions between humans and pigs. Such models, however, generally do not simulate neurocysticercosis itself or associated symptoms. CystiHuman complements these efforts by proposing a model of neurocysticercosis, associated symptoms, and likelihood of treatment. Overall, initial model outcomes are coherent with field data regarding neurocysticercosis prevalence and age patterns. The model suggests that short-term interventions that successfully reduce parasite transmission to a small fraction of its original level, but are followed (as has often been the case so far) by a rapid recuperation in transmission levels, will likely fail to produce a substantial reduction in neurocysticercosis prevalence. We view the present model as a first iteration of CystiHuman. Future iterations should use new field data to refine aspects of the model and ensure its full validation. They should also include cost information to inform cost-effectiveness analyses of interventions to address the disease.

## Introduction

Cysticercosis is a neglected tropical disease affecting humans and pigs, and a major cause of epilepsy in developing countries [1, 2]. Eating undercooked meat from pigs infected with cysticercosis can lead to human intestinal infection with the adult *Taenia solium* parasite; this infection is called taeniasis. Meanwhile, pigs eating *Taenia* eggs or proglottids can develop the larval stage of the parasite in the body where it forms cysts, leading to what is called cysticercosis. Open defecation and free roaming of pigs promote contacts between pigs and *Taenia* eggs/proglottids hence the spread of the parasite. Humans can also accidentally ingest *Taenia* eggs through the fecal-oral route, which may result in human cysticercosis. Human cysticercosis can have significant health effects in humans, particularly if cysts develop in the central nervous system (leading to neurocysticercosis, or NCC), which can lead to multiple presentations including epilepsy, migraine, intracranial hypertension (ICH), hydrocephalus and even death [3–5], for an estimated 2.8 million disability adjusted life years (DALYs) lost [6].

The World Health Organization has increasingly called for interventions to control or eliminate *T*. *solium* transmission [2]. While evidence on the effectiveness of interventions in reducing transmission between humans and pigs has been building [7–10], key information needed for policy development remains largely unavailable, including the effect of interventions on reducing the burden of NCC in the population. This gap is largely due to the costs and timeframes associated with neuroimaging needed to measure this burden over a large scale and over the many years needed before significant reductions in NCC are seen. In this context, computer simulations of the disease (based on models requiring neuroimaging data only for calibration/validation), combined with existing field tests, can provide more confidence regarding the best interventions to implement in different contexts. So far, multiple papers [11–16] have modelled the *Taenia solium* transmission cycle and some have modelled the number of NCC cases [11, 14, 15]. However, none have modelled the course of the disease or its symptoms.

We developed an agent-based model (ABM), CystiHuman, to address this gap. The novel features of this model include: 1) simulation of ICH/hydrocephalus (grouped within a single category for the simulation of both symptoms and treatment, for simplicity purposes) and epilepsy, including prevalence, treatment, and mortality, 2) differentiating model outputs according to the location of the lesion: parenchymal or extra-parenchymal (the latter being generally associated with the most severe symptoms) and 3) accounting for the time lag between infection and the appearance of symptoms/treatment, as well as time with the disease. Further, with CystiHuman, it will be possible to compare the cost-effectiveness of a large array of interventions, from taeniasis or cysticercosis treatment, to interventions to mitigate NCC through improved diagnosis and symptom management, which cannot be simulated using transmission models.

The objective of this paper is to describe this new model, including its purpose, scope, processes, and the information used to guide its development. We also explore the extent to which the model can be calibrated to real-world data collected from a variety of sources, and then apply the model to an endemic village in Northwestern Peru [17] to compare model outputs to observed data from that village. Model development is an iterative process that responds to both new data and knowledge from the real world, as well as to increased understanding of the model itself and of its performance. We consider this the first iteration of CystiHuman. Future versions will include more in-depth analysis of the behavior of the model, analysis of the impact of interventions on disease prevalence and their economic and DALY costs/benefits, and results from field studies that should provide data to refine the model and/or validate certain of its aspects.

## Methods

### Purpose

We developed CystiHuman with the long-term goal of informing decision-making through a cost-benefit analysis of different interventions to address neurocysticercosis. This paper focuses on how we model the prevalence and symptoms of human NCC, treatment likelihood, and symptom evolution. The primary model outputs are average NCC prevalence, person-weeks with different symptoms or treatments, and the number of surgeries and deaths.

Data are currently insufficient to validate the model, but comparison of model simulations with available field data can nevertheless be useful while awaiting for the results of planned field studies. For that purpose, we applied the model to an endemic village in Northwestern Peru, Rica Playa [17], and compared simulated and actual values in this village.

### Prerequisites

CystiHuman requires input from a separate model of the human taeniasis-pig cysticercosis cycle that gives information on the evolution of taeniasis and *Taenia* egg density in the community. We have chosen an adaptation of the CystiAgent model [16] for this purpose, as it provides the inputs needed and was validated in Peru, where our team has been working. Eggs in the environment, originally represented as a number in [16], are now represented as a density. Information regarding adaptations to CystiAgent relevant to CystiHuman since the original paper [16, 18] is provided in S1 Text.

CystiHuman also integrates demographic movements from CystiAgent. These include short-term mobility (travels to and from other villages), and long-term ‘mobility’ (emigration, deaths, immigration and births by age range), with immigrants differentiated according to their origin (high risk endemic area or low risk area). Integration of such movements is necessary, as Peruvian society is highly mobile, with migrations into and out of districts in the target region estimated at over 3% of the district population per year [19–21].

Finally, CystiHuman uses the same human and household allocation as CystiAgent, which reflects the actual situation in the target villages.

### State variables and processes

The model works at multiple levels: each village is a collection of households, which contain human individuals, who may host NCC lesions.

*NCC lesions* are individual agents located within human hosts. Multiple NCC lesions can be simultaneously present in a given host, modelled as independent agents. Each NCC lesion has seven state variables. Lesion-related processes include: 1) change in the stage/substage of the lesion (based on its age and in some cases treatment type), and 2) association with symptoms, which updates the state variables ‘time since last seizure’ and ‘association with ICH/hydrocephalus’. In the model code, cyst-related processes are implemented before human-related processes.

*Humans* are the second class of agents. They may harbor any number of NCC lesions, and are located within a village household (except for emigrants). They have nine state variables. In the current form of the model, there is no distinction between sexes (behaviors may differ by sex, but field data suggest that men’s and women’s taeniasis rates are similar, reducing the relevance of sex-disaggregation) and immunity to cyst development has been neglected, though these are features that could be added at a later stage of the model. Human-related processes include: 1) infection and cyst initiation through egg ingestion, 2) symptom development, 3) treatment, including type, delay and success and 4) travel, emigration and deaths.

*Households* belong to a specific village in which they have a fixed location in line with their actual location in the field. All households contain at least one human agent, while *villages* are open systems with in and out movement of humans, but a fixed number of households. Human agents do not change household within the village. Household related processes are limited to the replacement of departing humans with immigrants or newborns so as to keep overall population size constant, and to the welcoming of short-term travelers from villages outside of the simulation. Replacement of departing humans is implemented as humans emigrate or die naturally or through disease (details and justifications in S3 Text).

NCC lesions directly affect human hosts as their state determines the host’s disease and symptoms e.g., likelihood of seizure. Hosts may affect NCC lesions through treatment (e.g., surgical removal of a cyst). Humans interact with one another: an infected cook may affect household members through the preparation of contaminated food; while humans indirectly affect others from the same or other households in the village through environmental contamination. This indirect interaction between households is the only such interaction represented within CystiHuman. Meanwhile, in the absence of sufficient data to support the existence of interactions between lesions or represent them, we make the simplifying assumption that NCC lesions do not interact with one another. This assumption may be adjusted if more data become available in the future. The model does not include adaptive responses beyond humans’ choice to get treated or not after symptomatic disease appears.

The model has a spatial structure. In addition to interactions of humans within households, the underlying model of human taeniasis (CystiAgent) and some of the interventions (e.g., ring strategy) we want to assess are spatial in nature. Spatial location is modelled through a discrete location variable (latitude and longitude) on a square lattice. When the model is initiated i.e., at the beginning of the burn-in period, humans have no cyst in their encephalus. When cysts are created, they are immediately allocated a location, values for *τ*_*1*_ and *τ*_*2*_, immature stage, and association with no symptom. The immature stage of a lesion has a fixed duration, but the duration of other stages and all other processes are stochastic, as stochasticity is key to capture the time spread in symptom emergence.

Finally, the model is divided into three modules: module 1 simulates NCC prevalence and cyst stage; module 2 epilepsy/seizures; and module 3 ICH/hydrocephalus. The modules are calibrated successively. For the calibration of modules 1 & 2, extra-parenchymal lesions are ignored because they are rare among all lesions and epilepsy cases (see S1 Data). For module 3, they are included as they drive most ICH/hydrocephalus cases.

The description of state variables, their meaning, possible values and initial values are provided in Table 1.

**Table 1:** State variables for individual NCC lesions, human agents and households, and initial values.

Variable names (units)	Possible values (specifying if static)	Meaning	Initial value
**Variables for individual NCC lesions**			
Age (in weeks)	Positive integers	Time from inception of the lesion.	0
Parenchymal location	Yes/No (static)	Location of the lesion: it contributes to determine the likelihood and severity of symptoms associated with the lesion.	Random allocation based on the rules of the model (see “sub-models”)
Stage	Immature, mature non-calcified, calcified, disappeared	Describes the lifecycle of the lesion. Different stages and substages are associated with different likelihoods of symptoms. The immature stage is invisible on imaging. “Disappeared” is also invisible and was introduced solely for modelling purposes.	Immature
Substage length *τ*_*1*_ (in weeks)	Positive integers (static)	Duration of the first substage within the mature non-calcified stage, an asymptomatic period for all lesions. During this asymptomatic substage, the lesion is often (though not always) viable. The definition of this stage is not based on what is seen on imaging but on symptomatology, though the two often coincide.	Random allocation based on the rules of the model (see “sub-models”)
Substage length *τ*_*2*_ (in weeks)	Positive integers (static)	Duration of the second substage within the mature non-calcified stage (for parenchymal lesions). This substage may be symptomatic, and often corresponds to a degenerating lesion.	Random allocation based on the rules of the model (see “sub-models”)
Time since last epileptic seizure *t*_*seizure*_ (in weeks)	Positive integers or NA	This helps keep track of the time since the last epileptic seizure. It is formally, for the purpose of the model, associated with the individual lesion. In reality, clinicians can rarely identify one specific lesion as the origin of a seizure. However, patients with epilepsy tend to have more lesions (52.5% have multiple lesions [22–27]) than asymptomatic patients (27.5% have multiple lesions [17,22,28–32]), suggesting that seizure risk increases with the number of lesions, a specificity that is better modelled by associating an additional risk to each additional lesion rather than a flat risk linked to the host’s NCC status.	NA
Association with ICH and/or hydrocephalus	Yes/No	Whether the lesion is associated, or not, with increased pressure (ICH) and/or cerebrospinal fluid accumulation (hydrocephalus) in the encephalus.	No
**Variables for human agents**			
Age (in weeks)	Positive integers	Age of the human.	In line with village demographics (as in CystiAgent)
Household	ID	Household the agent belongs to.	In line with village demographics (as in CystiAgent)
Cook	Yes/No (static)	The human is responsible (or not) for cooking food for the household. There is exactly one ‘cook’ in each household. A cook with taeniasis may infect household members through contamination of prepared food with eggs.	Random (one per household)
Taeniasis status	Yes/No	Presence/absence of a mature taenia.	Randomly assigned at baseline as per CystiAgent
Epilepsy status	Active epilepsy, inactive epilepsy, asymptomatic	Epilepsy is defined as at least 2 unprovoked seizures at least 24 hours apart, while active epilepsy is defined as a person with epilepsy having had at least one seizure in the past 5 years [33]. Conversely, inactive epilepsy refers to a case in which the latest seizure took place over 5 years ago. Asymptomatic individuals are defined as individuals that do not have epilepsy (active or not).	Asymptomatic (no epilepsy)
ICH/hydrocephalus	Yes/No	The human host has ICH/hydrocephalus if at least one of its NCC lesions is associated with these pathologies.	No
Epilepsy treatment status	Current/Past/Never	Whether the patient receives or received treatment for epilepsy.	No
Epilepsy treatment success	Yes/No	Whether epilepsy treatment (if implemented) will be successful (no seizure) or a failure (breakthrough seizures). Individuals are randomly assigned to treatment success with probability *r*_*success*_.	Yes with probability *r*_*success*_, No otherwise
ICH/hydrocephalus treatment	No, non-surgical, surgical	Type of treatment received for ICH/hydrocephalus, if any. Non-surgical typically corresponds to anthelminthic treatment.	No
ICH/hydrocephalus treatment delay (in weeks)	Positive integers or NA	Time between first ICH/hydrocephalus symptoms and treatment (there is often a significant delay between the start of symptoms and diagnosis/treatment).	NA
**Variables for households**			
Location	Discrete latitude and longitude coordinates	Household location (fixed).	In line with actual household locations within the village

### Sub-models

The following section details the three main modules of CystiHuman: prevalence, epileptic symptoms, and ICH/hydrocephalus symptoms.

#### Module 1: NCC prevalence (infection risk and cyst stages)

Module 1 concentrates on disease prevalence, ignoring disease symptomatology. It assumes that extra-parenchymal lesions represent a small enough share of all lesions (when asymptomatic parenchymal lesions are included) to be ignored for the purpose of the module. NCC prevalence is determined by two different processes, infection risk and cyst stages:

#### Infection risk

The likelihood of developing cysts in the encephalus is determined by three main drivers:

- Self-infection risk: the likelihood of self-infection of a person with *T*. *solium* taeniasis, in the absence of protective hygiene practices (e.g., hand washing), is characterized by *χ*.
- Infection of household members by a person with *T*. *solium* taeniasis: this happens primarily if the person responsible for food preparation has taeniasis and limited hygiene. Household members of a person with taeniasis identified as a cook have an added infection risk noted *a χ* with *a ≤ 1* (assuming that the risk of contamination when eating food prepared by an infected cook is equal or lower than self-contamination risk when one has taeniasis).
- Risk of contamination through disseminated eggs in the overall environment. If *E* is the average density of eggs in the environment, the likelihood of environmental infection, if hygiene is poor, *E* is assumed to be uniform at village level and is noted *σ E*. For large communities, this assumption will likely no longer be valid and we may use a function *σ f(E(location))*, with *E(location) = egg density*, *f* being a bell-shaped function centered on the individual’s household and reflecting the places where s/he is typically present.

All infection risks are modulated by hygiene, represented by a multiplicative term *h* (*h = 1* if there is no hygiene, *h = 0* if hygiene is fully protective).

Simultaneous development of multiple cysts is not impossible. To account for this risk, the weekly risk of developing *k* cysts is given by a Poisson distribution with *λ = h (σ E + χ)* for a person with taeniasis, *λ = h (σ E + a χ)* for non-infected household members of an infected cook, and *λ = h σ E* otherwise.

**Fig 1:**
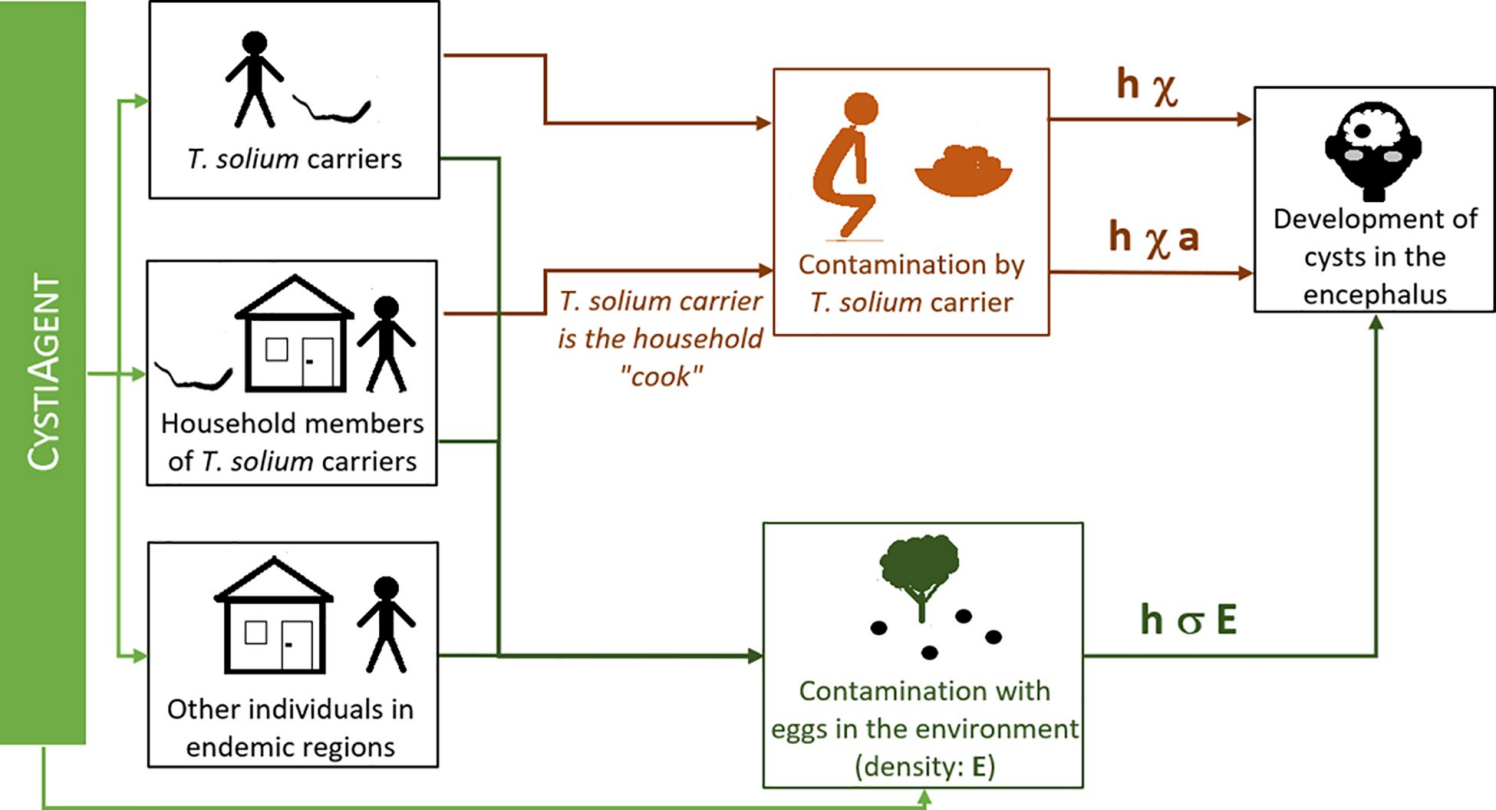
Risk of developing cysts in the encephalus following egg ingestion.

The way infection risk is modelled in CystiHuman and how this links to the outputs of CystiAgent (taeniasis cases and eggs in the environment) is detailed in Fig 1.

#### Cyst timeline

Once a cyst develops in the encephalus, it goes through multiple stages: starting off as an immature cyst for duration *τ*_*0*_ *= 3 months* [34], it continues as a mature non-calcified lesion, then either calcifies with probability *p*_*calc*_ or disappears. The mature non-calcified stage is divided into two substages, a first asymptomatic substage of duration *τ*_*1*_, and a second substage of duration *τ*_*2*_ that may be symptomatic. These substages are not directly related to changes in what is seen on imaging but to symptomatology, even though the two may coincide (see Modules 2 and 3).

### Module parameters

Table 2 provides the detail of known parameters in all three modules. There are three unknown parameters in module 1 that will be determined through the calibration process: *h* σ, *h χ* and *a*. *h* was introduced to highlight the contribution of behavioral drivers (*h*) vs. biological drivers (*χ*, *a* & σ), but does not need to be separated from these to accurately simulate NCC in the village. S1 Text details how cyst lifecycle indicators and indicators associated with disease prevalence and cyst number were computed.

**Table 2:** Values of the different parameters.

Parameter	Symbol and value
**Known parameters** *	
Delay from infection to mature cyst	*τ*_*0*_ *= 3 months* i.e., 13 weeks (fixed) [34]
Period from cyst maturity to lesion disappearance or calcification (*τ*_*1 +*_ *τ*_*2*_, driven by a 2-part distribution)–parenchymal lesions only	*τ*_*1*_ (duration of the 1^st^ substage) is given by a Gamma distribution: $Probability\;of\;\tau_{1}={\left(\tau_{0}+\tau_{1}\right)}^{\alpha -1}\;\beta^{\alpha }\;e^{-\beta (\tau_{0}+\tau_{1})}\frac{1}{\Gamma (\alpha )}$ with all times in years and *α = 2*.*94*, *β = 0*.*83*, then taking the maximum of the resulting τ_1_ and *1 week* to avoid *0* or negative values. *τ*_1_ derives from the analysis of estimated timeframes from infection to first symptoms in [35]^†^. *τ*_*2*_ (duration of the 2^nd^ substage, from the end of the 1^st^ substage to cyst death), is given by an exponential distribution which parameter is the average weekly death rate of a cyst: 2.6% (plausible range: 1.8–3.8%). See [36–45]^†^ and S1 Text
Probability for a parenchymal lesion to calcify vs. resolve without calcification	*p*_*calc*_ *= 32%* (plausible range: 25–38%)–see [36–42,44–46]^†^ and Fig D in S1 Text and S1 Text
Probability of seizure recurrence after disappearance of a parenchymal lesion associated with epilepsy	*s* = 10%—see [39, 40]^†^ and S2 Text
Probability of seizure recurrence within 3 years (or more) of antiepileptic treatment	*r*_*success*_ = 46.7% [38.6%-54.4%] [47]^†^ - see details in S2 Text
Treatment gap for active epilepsy (% untreated among those needing treatment)	*g*_*e*_ = 75%—based on [48] and dataset in S1 Data^†^
Case fatality rate (per year) for individuals with NCC-related active epilepsy	*d*_*ae*_ = 0.138% [0.069–0.584%] [33]
Weekly likelihood for unsuccessfully treated or untreated calcified parenchymal lesions associated with epilepsy to be associated with a seizure	*ω* = 0.05 –see explanation of the computation of this figure in S2 Text
Delay from cyst maturity to first symptoms, for extra-parenchymal lesions	*τ*_*1*_ (duration of the 1^st^ substage) is given by a Gamma distribution: $Probability\;of\;\tau_{1}={\left(\tau_{0}+\tau_{1}\right)}^{\alpha -1}\;\beta^{\alpha }\;e^{-\beta (\tau_{0}+\tau_{1})}\frac{1}{\Gamma (\alpha )}$ with all times in years and *α = 17*.*21*, *β = 1*.*10*, then taking the maximum of the resulting *τ*_*1*_ and *1 week* to avoid *0* or negative values. τ_1_ derives from the analysis of estimated timeframes from infection to first symptoms in [35]^†^.
Delay from first ICH symptoms to treatment	*t*_*delay*_: 37% of cases delay treatment by less than 1 month, 36% by 1–6 months, 10% by 6–12 months, and 19% by over 1 year–see [49–53]^†^ and S2 Text and Table C in S2 Text
Probability of treatment, per treatment type, for ICH/hydrocephalus	- No treatment: notreat = 90%-100% (95% used in simulations) Medical treatment only, nosurg = 20% of treated patients. Surgical treatment (e.g., cyst excision, shunt): 80% of treated patients–see [50, 54, 55]^†^ and S2 Text
Average number of surgeries/shunt revisions per surgical case	- *N*_*surgeries*_ = 1.2 (plausible range: 1.0 to 2.3) per surgical case—see [49, 53, 56–62]^†^ and S2 Text
Case fatality rate (total) for individuals with NCC-related ICH/hydrocephalus	- Medical treatment only: simplified to 0% (failed treatment followed by surgery falls under “surgical treatment”). - Surgical treatment: dsurgery = 18.3% [15.4–21.2%] in 2000, with a 3.6% [2.5–4.7%] decrease in risk per decade e.g., death rate = 11% in 2020 and 36% in 1950, 10% risk today [49, 56, 57, 63–66]^†^ & Figs A and B in S3 Text and S3 Text - No treatment: duntreated: at least 36% (rate for treated cases in the 1950s).
**Tuning parameters**	
Risk of self-contamination for a *T*. *solium* tapeworm carrier	*h χ*
Risk of contamination of household members by a *T*. *solium* tapeworm carrier that prepares household food	*h χ a*
Coefficient reflecting the strength of environmental contamination risks	*h σ*
Probability for a single parenchymal lesion to be associated with epilepsy at the mature non-calcified stage	*π*_*e*_
Probability for a single parenchymal lesion to be associated with epilepsy at the calcified stage	*π*_*ec*_
Share of individuals with epilepsy at the mature non calcified stage that continues having active epilepsy at the calcified stage	*π*_*ae*_
% of new lesions that are extra-parenchymal**	*Ξ*
Likelihood of ICH associated with a single parenchymal lesion	For non-calcified mature lesions: *π*_*i*_

* All parameters representing time are ultimately expressed in weeks, and rounded to the nearest integer.

** Ignoring extra-parenchymal lesions that are asymptomatic during their whole lifespan.

^†^ Value not directly taken from the reference but obtained through process explained in S1 Text, S2 Text or using data in S1 Data.

### Module 2: epilepsy symptoms, treatment likelihood and risk of death

Module 2 simulates epilepsy symptoms, treatment, and risk of death. It only includes parenchymal lesions as the large majority of epilepsy cases are associated with such lesions. The rationale for all figures is detailed in S2 Text while mortality data are provided in S3 Text.

#### Likelihood of symptoms associated with a parenchymal lesion

parenchymal lesions may be associated with incident epileptic seizures at the beginning of the second substage of the mature non-calcified stage (with probability *π*_*e*_) or at the beginning of the calcified stage (with probability *π*_*ec*_). When cysts that were associated with seizures prior to calcification reach the calcified stage, associated seizures may stop or continue (the corresponding probability is noted *π*_*ae*_). Meanwhile, the model defines a probability of seizure in any given week for calcified lesions associated with active epilepsy in patients that have never been treated (or have been unsuccessfully treated) as *ω*. This representation simplifies active epilepsy as it does not represent individuals with highly irregularly spaced seizures.

Finally, if lesions that have already been associated with epileptic seizures disappear, further seizures may take place before waning: the corresponding probability is *s = 10%*. In such cases, it is assumed, as a simplification, that new seizures happen at the moment of lesion disappearance but stop afterward.

#### Human symptoms

symptoms for a human host are derived from the symptoms associated with individual brain lesions: if any of the brain lesion in the model has been formally associated with epilepsy (time since last seizure ≥ 0), the host has epilepsy. Humans have active epilepsy if the most recent seizure associated with any of the host’s lesions happened less than *T*_*a*_ = 5 years or 261 weeks ago. This value was chosen in line with practices in the region of Peru to which the model has been applied.

#### Treatment

treatment with anti-epileptic medication may be undertaken if the individual has epileptic seizures. The model uses the estimated probability of treatment in endemic communities in the target region of Peru. It assumes that treatment, once initiated, continues for a duration *T*_*treat*_ after the last seizure. Based on feedback from Peruvian colleagues, we used *T*_*treat =*_ *2 years*, but leave space for other options as needed.

Treatment is deemed “successful” if seizures stop while the patient is being treated (no “breakthrough seizures”). Treatment success leads to the end of treatment after the patient has remained seizure free for at least two years. Treatment success is most relevant at the calcified stage as drugs are normally not discontinued during the shorter non-calcified stage [4]. The probability of success (cessation of seizures) when calcified lesions are treated with anti-epileptic medication for two years has been estimated at around 47% [47]. In the model, patients that have been successfully treated no longer experience seizure, even after treatment is discontinued. When treatment is not successful, patients are modelled as having continued seizures both during and after treatment at frequency *ω*. We do not model the variety of situations among “unsuccessfully treated” patients, some of whom may have significantly reduced seizure frequency.

#### Mortality

deaths from active epilepsy are rare. These are computed based on Peru’s data. For simplicity reasons, individuals that die from active epilepsy are replaced by immigrants or births in the same household, in line with the rules of the demographic model.

### Module 3: extra-parenchymal lesions, ICH/hydrocephalus, treatment likelihood, timing and type, and risk of death

Module 3 introduces extra-parenchymal lesions. Though rare in population surveys, they are often associated with the worst disease course (ICH or hydrocephalus and death) hence are important to compute the burden of NCC. *ξ* % of all lesions are extra-parenchymal. Since such lesions are important, not because of their number but because of the severe symptoms they create, any extra-parenchymal lesion which would never create symptoms or would solely be associated with epilepsy is ignored in the model.

#### Stages of extra-parenchymal lesions

extra-parenchymal lesions are assumed to reach maturity at the same speed as parenchymal lesions. On the other hand, *τ*_*1*_, the time from maturity to first symptoms, is much longer than for parenchymal lesions [55]. The calcified/disappeared stages and the possibility of spontaneous long-term cure are not included in the model for extra-parenchymal lesions.

#### Symptoms

all extra-parenchymal lesions represented in the model are associated with ICH/hydrocephalus, starting at time *τ*_*0*_ *+ τ*_*1*_. Parenchymal lesions are rarely associated with ICH/hydrocephalus, but if they do so this also happens after *τ*_*0*_ *+ τ*_*1*_ and only at the non-calcified stage. The associated probability is noted *π*_*i*_.

*ICH/hydrocephalus treatment* is very rare in the context of endemic Peruvian villages, though the exact share that gets treated is unknown. In addition to people that never get treated, many of those who ultimately consult a doctor delay care-seeking. Treatment, when it takes place, may be non-surgical (primarily anthelminthic) or surgical (shunt placement, cyst excision, etc.). Death rates are elevated, and primarily known for individuals that do seek treatment. It can be assumed that they are higher for those that do not.

Table 2 provides module parameter values, how they were obtained is detailed in S2 Text (for symptoms) and S3 Text (for deaths).

**Fig 2:**
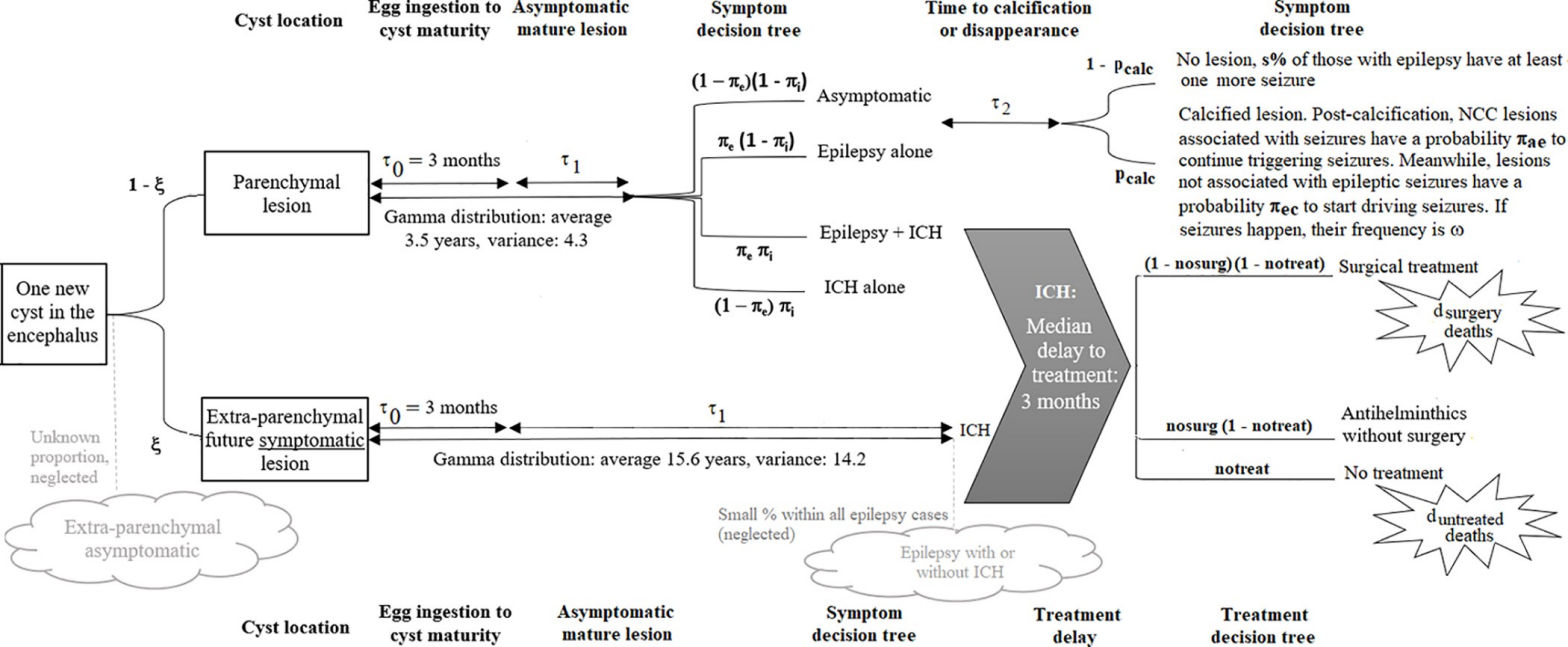
Timeline and symptoms associated with an NCC lesion.

Fig 2 provides a graphical representation of the progression of lesions through different stages, and the likelihood of symptoms and treatment. This information is used in all 3 modules. For simplicity of the graphical representation, the likelihood of treatment and risk of death from epilepsy have not been represented on the graph though they are included in the model. Further, the word “ICH” has been used to refer to ICH or hydrocephalus. Flow charts closely reflecting each of the steps taken by the model code are provided in Figs A, B and C in S4 Text.

### Programming and implementation

The model was programmed in Java MASON and the corresponding code (as well as the code for CystiAgents) is available at https://github.com/oflixs/CystiAgents.

The model is implemented using a burn-in period (during which statistics are not recorded) of 3,500 weeks (67 years) or roughly a human lifespan to allow for the accumulation of calcified lesions in all individuals in the modelled community. Statistics are then accumulated over 10,000 weeks (192 years) to produce baseline figures. We expect that the impact of control interventions will generally be assessed over one or several decades. NCC symptoms evolve over months and years, but taeniasis infections and environmental contamination with eggs, which drive NCC infections, evolve over the course of weeks and months, hence the model has a discreet time step of one week.

### Calibration methods

Model calibration serves to identify the values of unknown parameters (called “tuning” or “calibration” parameters) that lead to model outputs that best fit observable data. CystiHuman was tuned using multi-stage approximate Bayesian computation calibration. To do so, CystiAgent, the transmission model that has been chosen to provide inputs to CystiHuman, first needs to be calibrated. However, so far, there are no villages in which local contemporaneous data sufficient to calibrate both CystiHuman and CystiAgent are available. Field studies are planned to gather a comprehensive set of data for both models in the same community. In the meantime, we have chosen to tune CystiHuman in the same 3 endemic villages in North-West Peru, denoted as 515, 566 and 567, in which CystiAgent was calibrated [unpublished results], because data on village demographics, human taeniasis and pig cysticercosis are sufficient to fully calibrate CystiAgent there.

CystiHuman was tuned in three steps, corresponding to each of the three modules: calibration of *h σ*, *h χ* and *a*, which allows for the simulation of disease prevalence and stage (module 1), calibration of *π*_*e*_, *π*_*ec*_ and *π*_*ae*_, related to epilepsy and seizure symptoms (module 2), and calibration of *π*_*i*_ and *ξ*, associated with ICH/hydrocephalus symptomatology (module 3). For the calibration of modules 1 & 2, extra-parenchymal lesions are ignored because they are rare among all lesions and epilepsy cases (see S1 Data). For module 3, they are included as they drive most ICH/hydrocephalus cases.

The observables chosen to calibrate the model are presented in Table 3. The table provides the geographic origin of the data/proxies, while S1 Text (for module 1) and S2 Text (for modules 2 and 3) detail how the corresponding values were obtained. The second and third observables in the table (share of individuals with NCC that have a single lesion, and share having two lesions) are based on global averages as a review of literature has revealed that these shares were largely stable across countries and communities (see S1 Text, Fig A and Table A in S1 Text).

**Table 3:** Data used in to calibrate the model.

Module	Observable	Value
1	Prevalence of calcified NCC lesions (with or without other lesions) within the adult population	20% for similar endemic communities in Peru–see [17] and S2 Data
1	Share of individuals with NCC that have a single lesion	72.5% (95% CI: 66.6–78.4%) global average, see [17, 22, 28–32] ^†^ and Fig A in S1 Text
1	Share of individuals with NCC that have two lesions	16.9% (95% CI: 11.7–22.0%) global average, see [17, 22, 29, 30] ^†^ and Table A in S1 Text
2	Added lifetime epilepsy prevalence associated with neurocysticercosis	2.10% [1.05–3.69%], estimated using Latin America figures, see [2, 67] ^†^ & S1 Text
2	Share of never-treated calcified parenchymal NCC cases with NCC-driven epilepsy that have “long-term” active epilepsy	66.2% [53.7–77.2%] for North-West Peru–see S2 Text and dataset in S1 Data
2	Share of never treated NCC cases with active epilepsy and solely parenchymal lesions that have at least one non-calcified lesion	15.9% [10.3–23.1%] for North-West Peru–see S2 Text and dataset in S1 Data
3	Share of all NCC cases that have extra-parenchymal lesions in Peru (at community level)	Likely between 0.3% and 5% based on field data from Peru (see S2 Text for details). To be improved through field studies. 3% selected for this specific calibration.
3	Share of all clinical cases with ICH or hydrocephalus that have parenchymal lesions only	8.6% [0.0–17.3%] [54, 68–71]^†^, global average

^†^ Value not directly taken from the reference but obtained through process explained in S1 Text, Fig A Table A in S1 Text, S2 Text, S1 and S2 Data.

The three modules were calibrated successively. For the first two modules, the program explores a series of parameter sets at each stage of calibration. Multiple runs are simulated for each set, and outcomes averaged over all runs. The number of runs was chosen to limit uncertainty on the average found for each parameter set, while the number of parameter sets was defined to optimize the speed of convergence of the calibration, given the number of runs. A threshold below which a parameter set is accepted was also defined. This threshold was set so that around 20 parameter sets were accepted at each calibration stage. Table 4 provides details for the first two modules. The two unknown parameters in the third module can be calibrated successively, allowing us to use manual calibration. However, given the small number of cases in any single village, we had to average outcomes over 64 runs for each parameter set.

**Table 4:** Calibration details for the first two modules.

Module	Parameter sets	Runs	Threshold	Parameter space in first stage of calibration
1	3000	16	0.6%	[0–0.007] for *σ*, [0–0.06] for *χ*, and [0–1] for *a*
2	2000	32	0.9%	[0–0.06] for *π*_*e*_, [0–0.06] for *π*_*ec*_, and [0–1] for *π*_*ae*_

The ‘best’ parameter set is the one whose output is closest to the observed values used to calibrate the model. The distance *D* between simulated and observed values has been defined as Euclidean distance, scaled using the value of the observable. *D* is computed for each village then summed over all villages (with a weight proportional to village size), except for the share of adults with calcified NCC, for which we expect the average over all villages, rather than values in individual villages, to be close to the average value observed in other communities in the same region. Hence:

$$D^{2}=\sum_{parameters}D_{parameter}^{2}\;with$$

$D_{parameter}^{2}=\sum_{villages}\frac{p_{village}}{p_{total}}\frac{{\left({simulated}_{village}-observed\right)}^{2}}{{observed}^{2}}$, except for adults with calcified NCC:

$$D_{share\;of\;adults\;with\;calcified\;NCC}^{2}=\frac{{\left({average\;simulated}_{all\;villages}-observed\right)}^{2}}{{observed}^{2}}$$

Once actual values for NCC prevalence in the villages in which the calibration is undertaken become available, the calibration will seek to approximate these values for each village separately. The main results of the calibration process are described in the next section while further details are available in S4 Text.

## Results

This section includes a description of calibration results for villages 515, 566 and 567 in the Piura region of Peru. It also describes initial model outcomes (e.g., age-related patterns) and applies the model to a fourth village: Rica Playa. The purpose of this section is to: 1) demonstrate the feasibility of model calibration using available data, and 2) provide and discuss initial model outcomes based on this calibration. Field studies are planned to improve model observables hence calibration results and obtain data from further villages for validation. In-depth discussion of the model, description of the model of the costs of the disease and analysis of the impact of interventions, will be done in separate papers.

### Calibration results

The calibration significantly narrowed the range of plausible parameters for all parameters. Calibrated values are: *h σ* = 0.00215, *h χ* = 0.0180, *h a* = 0.0714. π_*e*_ = 0.0050, *π*_*ec*_ = 0.0132, π_*ae*_ = 0.406, *ξ* = 0.0108 and *π*_*i*_ = 0.00083. Simulated outcomes using these values yield outputs that are very close to the targets (see details in S4 Text).

The calibrated value for *h a* is small, corresponding to a strength of self-infection (*h χ*) substantially higher than that of contamination through cooks (*h a χ*): after a year with taeniasis, the risk of contamination is 61%, while after a year eating food prepared by a household member with taeniasis, the risk is 6%. There is also a 1.4% yearly contamination risk from environmental sources (a continuous, unavoidable source of infection, which magnitude is reflected by *h σ*). Over 50 years, this corresponds to one chance in two of being contaminated. Overall, using the selected calibration parameters, 42% of NCC cysts come from environmental contamination sources.

Meanwhile, the calibrated parameters for module 2 suggest that 0.50% of all lesions will be associated with epilepsy starting at the non-calcified (viable or degenerating) stage, while 0.42% (π_*ec*_ × *p*_*calc*_) will be associated with epilepsy starting at the calcified stage. Close to one in two lesions associated with seizures at the non-calcified stage continue being associated with seizures after calcification. In addition, when running the simulations, we find that close to 20% of all epilepsy cases caused by NCC are individuals who, on imaging, will not have any lesion, these epilepsy cases derive from a past infection in which the lesion has cleared. Once treatment likelihood is included, the model suggests that, for individuals in the target villages or who contracted the disease while there, over 10,000 weeks of simulation, 3.5 people on average will die from active epilepsy, 22.4 from ICH/hydrocephalus, and 2.6 will undergo treatment/surgery. The burdens of active epilepsy, treated active epilepsy and ICH/hydrocephalus over that period are 1231, 146 and 1731 person-years spend with the respective condition, assuming that the treatment gap is 95% for ICH/hydrocephalus and the death rate 36% for untreated individuals (the lowest estimate for that figure).

### Initial model outcomes and application to an additional village

**Fig 3:**
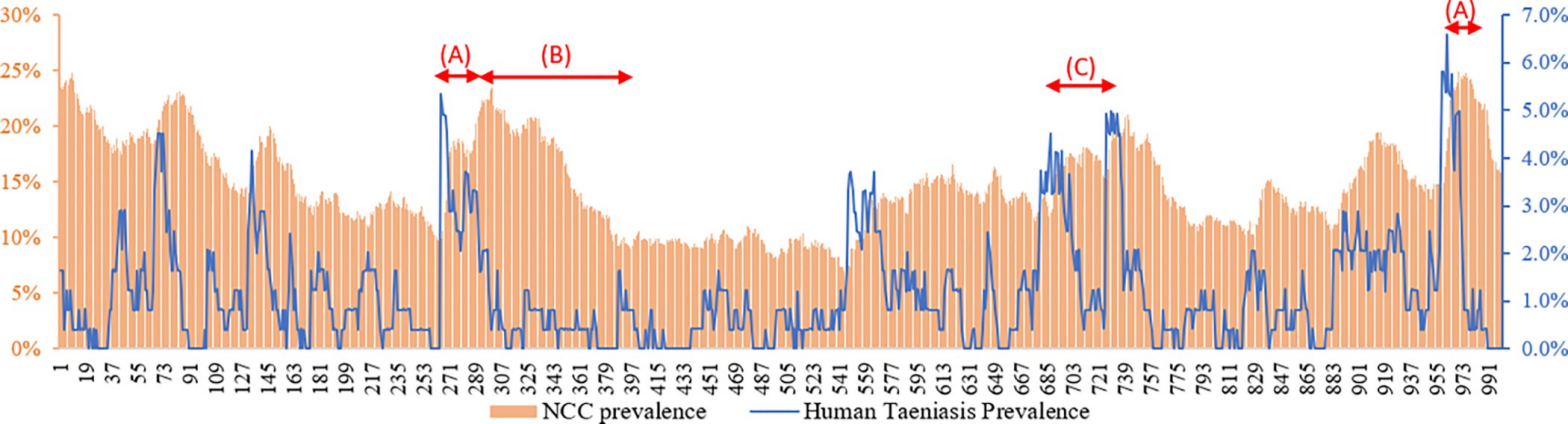
Links between variations in human taeniasis prevalence and variations in NCC prevalence. Note: the overall time scale is 192 years, or 10,000 weeks. One data point is represented for every 10 weeks (hence 1000 data points are represented here). The red arrows relate to specific zones of the graph that are discussed in the text.

In this section, we present some additional model outputs of interest. Fig 3 shows the evolution of NCC prevalence for the calibrated model for one run in one of the villages, and how this relates to oscillations in human taeniasis prevalence (in the absence of interventions). These patterns are similar for different runs and villages. Short peaks in taeniasis rapidly lead to an increase in NCC prevalence **(A)**, followed by a slow decrease **(B)**, over decades. Further, a rapid succession of peaks and lows in taeniasis prevalence is not associated with a substantial decrease in NCC prevalence **(C)**, which suggests that short-term interventions, if they are followed (as has been the case so far [9, 10]) by a rapid increase in taeniasis back to original levels (also associated with a rapid increase in pig cysticercosis), may have limited impact on NCC rates.

Data availability so far is insufficient to validate CystiHuman. However, CystiHuman can be applied to the Peruvian village of Rica Playa [17] and model outcomes compared with a number of field measures. Further, it is possible to compare model outcomes for the three calibration villages with a number of actual figures from other communities/contexts for which data are insufficient to apply the model, keeping in mind that CystiHuman results may not be fully transferable to these new contexts.

#### Age at first symptom

In model simulations, across the three villages, seizures are expected to begin at 32 years old while ICH/hydrocephalus symptoms should begin at 41 years old. Clinical data suggest that there is indeed a difference between these ages: in a review of 38 cases, the average age of patients with ICH/hydrocephalus was 39.6 while that of patients with epilepsy and no ICH/hydrocephalus was 33.3, a 6.3-year difference [54]. Further, multiple studies have compared the age of symptomatic patients with parenchymal vs. extra-parenchymal lesions (the first group mostly presented with epilepsy and the second with ICH/hydrocephalus): in these studies, patients with parenchymal lesions were on average 7.2 [70], 3.5 [69] and 2.3 [72] years younger than those with extra-parenchymal lesions.

#### Comparison of model outcomes with field data in Rica Playa [17]

We applied the calibrated CystiAgent and CystiHuman models to Rica Playa, simulating taeniasis, pig cysticercosis, human NCC prevalence and NCC symptoms. We defined a confidence interval in which 95% of experimental measures (should all the adult population be sampled), would fall. These results were compared with field study results, based on CT scans of 86% of the adult population. Results are available in Table 5. The only significant difference between simulations and actual figures relates to the number of cases with 11 or more lesions. There is good coherence between all other outputs and field measures. Note that the patterns of increase in NCC prevalence with age found in both simulations and field data from Rica Playa is also generally observed in other endemic communities e.g., in Mexico [32] and Ecuador [28]. Age-related increases derive from the accumulation of infection risks over time, and are likely to be influenced by phenomena such as acquired immunity and historical changes in pig raising practices or hygiene levels.

**Table 5:** Model outputs and observed values (where available)*.

Indicator	Model outputs	Observed value
Human taeniasis prevalence	3.0%	NA
Pig cysticercosis prevalence	3.1%	NA
Overall human NCC prevalence	16.5%	NA
% of adults with calcified NCC lesions	17.2% [9–26%]	18.8% [16.9–20.6%]
% of adult NCC cases with a single lesion	74.9% [61.9–87.7%]	66.7% [62–71%]
% of adult NCC cases with 11+ lesions	0.0003% [0–0%]	4.2% [3–7%]
Epilepsy prevalence among adult NCC cases	2.0% [0–7.0%]	0%
Estimated OR of having NCC (40–59 vs. 20–39 years old)	1.7 [0.8–3.6]	1.6
Estimated OR of having NCC (60+ vs. 20–39 years old)	2.5 [0.8–6.0]	2.5

* Note that the confidence interval for model outputs relies on the assumption that the temporal variabilities of CystiAgent and CystiHuman are accurate. However, it has not yet been possible to check this using experimental data.

It is important to note that Rica Playa [17] is one of the villages included, alongside other communities, in the computation of some of the proxies used when calibrating CystiHuman. This increases the likelihood that projections for this village would align with reality, though coherence is not a given. For example, the use of a global average for the shares of NCC cases with a single or two lesions in the three villages of calibration was premised on the assumption (supported by community data, as shown in S1 Text) that these shares are very stable across countries and communities. However, using these to calibrate CystiHuman did not guarantee that the model would lead to stable figures across communities, and it was reassuring to find that model simulations in Rica Playa were coherent with field data. To validate the model, however, there will be a need to apply it to a set of entirely new villages–something we are working toward through plans for new field studies.

## Discussion

The primary objective of this paper is to present CystiHuman, an ABM that simulates human NCC in the endemic community setting. This ABM represents an important first step toward filling a critical gap in the field of *T*. *solium* control and elimination, namely a tool that can simulate the prevalence and incidence of NCC, associated disease manifestations such as epilepsy and ICH/hydrocephalus, and their cost, to inform policy decisions. In this paper, we demonstrated that a functional ABM of NCC can be developed based on current understanding of the processes involved and on existing data sources. Furthermore, we showed that this model can be calibrated successfully to reproduce observed patterns of NCC in endemic villages in northwestern Peru, such as age-prevalence increases, despite employing calibration targets that were not specific to these villages. Finally, we showed that using these calibrated parameters, CystiHuman adequately reproduces real-life data observed in another rural village in northern Peru. However, more work is needed to achieve the goal of a credible model that can be used for policy decisions.

One of the main challenges in developing CystiHuman was the general paucity of real-world data to inform parameter estimates and processes used in the model. As is the case for most neglected tropical diseases, literature on NCC is limited due to a historic lack of attention, and funding, to the disease. Poor accessibility to neuroimaging in endemic regions, suboptimal performance of diagnostic assays [73], and lack of standard approaches to screening and diagnosis, further limit the scope and quality of the literature base. In general, available studies had small sample sizes [25, 27, 74–76], were cross-sectional in nature or with short-term or incomplete follow-up, and were biased towards enrollment and/or follow-up of symptomatic cases (among papers with data on the number of lesions in NCC cases, 18 focused on symptomatic cases vs. 6 on asymptomatic or all cases–these were used in S1 Text). Further, studies employed a variety of diagnostic methods including CT scan, serology or mixed criteria [77], complicating efforts to synthesize results and often leading to wide confidence intervals for parameter estimates. For some potentially important processes, such as human immunity, which may differ by exposure, age, gender or genetic background, currently available data were insufficient to include these processes in the current iteration of CystiHuman. Nonetheless, we found sufficient data to build and successfully calibrate the ABM using a combination of global and regional data. Some target values for calibration (e.g., proportion of NCC cases with a single lesion) were remarkably stable across many studies, while others (e.g., share of NCC cases with parenchymal lesions or with ICH) could be improved with additional high quality field studies.

We were also challenged to find adequate data sources for validation purposes, as studies measuring NCC, epilepsy and ICH, along with taeniasis and porcine cysticercosis, were rare. We were able to conduct an initial cursory comparison using observed data from the small village of Rica Playa and modeled values from CystiAgent. It is important to note that Rica Playa [17] is one of the villages included, alongside other communities, in the computation of some of the proxies used when calibrating CystiHuman. This increases the likelihood that projections for this village would align with reality, but coherence is not a given. For example, the use of a global average for the shares of NCC cases with a single or two lesions in the three villages of calibration was premised on the assumption (supported by community data, as shown in S1 Text) that these shares are very stable across countries and communities. However, using these to calibrate CystiHuman did not guarantee that the model would lead to stable figures across communities, and it was reassuring to find that CystiHuman reproduced this share as well as most other observed values in Rica Playa using the set of tuning parameter values that were not specific to this village. The one element CystiHuman did not reproduce is the high share of cases with large numbers of lesions. Though this discrepancy should not affect the ability of the model to simulate disease prevalence, it suggests that some real-life phenomenon has not been accounted for. This may be, for example, an additional modality of infection affecting a minority of cases, or perhaps, inborn and/or acquired immunity. We are currently planning additional community studies in northern Peru to collect the comprehensive datasets needed to conduct more in-depth analyses of the model’s behavior, and for full validation against a set of entirely new villages. These studies should include some behavioral data (e.g., social networks, open defecation or cooking roles or practices), linked to demographic data (sex, age), which may help assess how much these may influence heterogeneities in infection risk.

Further refinements to the processes included in this model should also be considered. For example, model performance might be improved by allowing some lesions to die rapidly, leading to early calcification and symptoms, rather than to require these to always go through a viable stage [34]. Data from India [35] suggest that few of the UK soldiers that contracted NCC in India immediately developed symptoms, but it has been suggested that disease manifestations may differ in Latin America, possibly because of mechanisms of immunity [78]. Hence, developing a plausible model of human immunity, albeit very complex, would be an important next step for CystiHuman. Other possible refinements include a model of contamination through communal eating places, interactions with extended family, or a more detailed representation of contamination through dispersed eggs in the environment (the present model assumes uniform risk at village level). As further data become available regarding associations between NCC and chronic severe headache, cognitive impairment, and other manifestations [79–81], these could be added to the model to more accurately capture the burden of disease.

Once CystiHuman has been validated in the context for which it has been developed, assessing how well it can be transferred to other settings will be essential. One of the strengths of the model is that some of its parameters and observables are expected to be valid in multiple contexts. Its calibrated parameters are mostly dependent on the biology of the disease and on hygiene practices at village level. These may therefore be transferrable to other endemic communities in Northwest Peru, where human, pig and worm genetics, as well as hygiene practices, are expected to be similar. The observables used for calibration are also mostly non-local, reducing expected measurement efforts to re-calibrate the model to new contexts. The primary exception is NCC prevalence, which is village-specific. Its measurement can be costly and logistically difficult in certain communities, particularly if we wish to transfer the model to poorer country contexts. This pleads for increased efforts to improve biological markers of the disease, which are cheaper and easier to implement than CT scans and MRIs.

We developed CystiHuman because we believe that such a model could add new insights to those brought by transmission models focusing solely on human taeniasis and pig cysticercosis. CystiHuman will have the ability to model the impact of a broader array of interventions than transmission models, e.g., free supply of anti-epileptic drugs. More and better simulations will require the development of estimates of the economic and DALY costs of the disease (with some economic estimates coming from planned field studies) and testing of new model elements.

In conclusion, CystiHuman presents an important first step toward accurate modelling of human NCC, which could bring useful insights into the relative effectiveness and cost of different interventions to address the disease. In addition to providing a different perspective on interventions that can be modelled through transmission models, CystiHuman also has the ability to include new interventions focused on NCC mitigation. More field studies and further model development and testing are planned to ensure that CystiHuman provides a fully reliable tool to study the disease.

## Supporting information

S1 Text Inputs to the model of lesion stages and neurocysticercosis prevalence. Table A in S1 Text: distribution of NCC cases per number of lesions. Table B in S1 Text: Weekly probability of death of an NCC lesion after the beginning of symptoms. Fig A in S1 Text: Proportion of all NCC cases that have a single lesion. Fig B in S1 Text: Incident cases as a function of the number of years between first symptoms and exposure, actual and projected. Fig C in S1 Text: Share of active or transitional lesions that have died (calcified or disappeared), according to the time elapsed since first symptoms or diagnosis, in months. Fig D in S1 Text: Share of lesions that calcify among lesions that either calcify or disappear.
(DOCX)

S2 Text Data used as inputs to model NCC symptoms (excluding deaths). Table A in S2 Text: CT scan assessment (NCC or not) and cause of epilepsy (NCC-related or not) at diagnosis. Table B in S2 Text: proportion of cases of ICH/hydrocephalus that have extra-parenchymal lesions, by country. Table C in S2 Text: Delay between first symptoms and treatment for patients with ICH or hydrocephalus that ultimately sought treatment. Table D in S2 Text: Number of surgeries per surgical NCC case & share of surgical NCC cases that are cured. Fig A in S2 Text: Share of individuals with a single lesion among symptomatic NCC cases.
(DOCX)

S3 Text Demographic data (including NCC-related deaths) and methodology. Table A in S3 Text: 2016 estimate of the case fatality ratios for active epilepsy in selected American countries. Table B in S3 Text Parameters describing short-term movements to and from the villages. Table C in S3 Text: Percentage of Peru and Piura population, by age range, that died or emigrated out of the country between 2011 and 2016. Table D in S3 Text: Percentage of Piura population, by age range, that changed district within Peru between 2011 and 2016. Table E in S3 Text: Estimated yearly rate of emigration out of Gates 2 villages (movement out of the village of origin but within the country). Table F in S3 Text: 2016 Piura population, by age range, that lived in another district or abroad in 2011, as a share of 2011 Piura population in age range. Table G in S3 Text: Estimated yearly rate of immigration into Gates 2 villages. Table H in S3 Text: Immigration into Piura: actual for 2011–2016 and revised values. Table I in S3 Text: Emigration from a Piura district: actual for 2011–2016 and revised values. Table J in S3 Text: Deaths (plus emigration to another country): actual for 2011–2016 and revised values. Table K in S3 Text: Newcomers–immigrants and births (number of individuals and share of all newcomers). Fig A in S3 Text: Percentage of deaths among surgical cases. Fig B in S3 Text: Percentage of deaths among severe cases. Fig C in S3 Text: Representation of the ‘reality’ of demographic changes. Fig D in S3 Text: Simplification of people’s history when they have lived in different places with various levels of risks before coming to the village. Fig E in S3 Text: Simplified representation of demographic changes.
(DOCX)

S4 Text Flow charts and calibration details. Fig A in S4 Text: Brain cyst time step. Fig B in S4 Text: Human time step (Module 1): development of new (immature) cysts. Fig C in S4 Text: Human time step (Modules 2 & 3). Fig D in S4 Text: parameter range post calibration (Module 1). Fig E in S4 Text: parameter range post calibration (Module 2).
(DOCX)

S1 Data Dataset for epilepsy figures (Tumbes region). (XLSX)

S2 Data Dataset for calcified NCC (Matapalo area villages). (XLSX)
