# Supplementary material for: CystiHuman: A model of human neurocysticercosis
Source: PLoS Comput Biol. 2022 May 19;18(5):e1010118. doi: 10.1371/journal.pcbi.1010118 (PMC9159625; doi:10.1371/journal.pcbi.1010118)
Supplement: S1 Text — Table A in S1 Text: distribution of NCC cases per number of lesions. Table B in S1 Text: Weekly probability of death of an NCC lesion after the beginning of symptoms. Fig A in S1 Text: Proportion of all NCC cases that have a single lesion. Fig B in S1 Text: Incident cases as a function of the number of years between first symptoms and exposure, actual and projected. Fig C in S1 Text: Share of active or transitional lesions that have died (calcified or disappeared), according to the time elapsed since first symptoms or diagnosis, in months. Fig D in S1 Text: Share of lesions that calcify among lesions that either calcify or disappear. (DOCX) [file pcbi.1010118.s001.docx]

Supporting information 1 – Inputs to the model of lesion stages and neurocysticercosis prevalence

This document details how the figures used to model neurocysticercosis (NCC) prevalence and the stages of NCC lesions were obtained. It also provides a few additional explanations regarding CystiAgent. More specifically, this document covers:

1. Prevalence figures for endemic communities in North-West Peru.
2. The share of NCC cases with one, two or more NCC lesions in community-level studies.
3. The length of the stages of NCC lesions.
4. The probability of calcification of a parenchymal cyst.
5. What we know (and do not know) about the stages of extra-parenchymal lesions.
6. What has been adjusted in CystiAgent since the 2020 publication by Pray et al. [1].

Points iii) and iv) are relevant both to the description of cyst stages and to that of symptoms.

# NCC prevalence figures for endemic communities in North-West Peru

Limited information is available on NCC prevalence for endemic communities in Peru. Two studies have been undertaken in North-West Peru: in the Rica Playa community [2] and the Matapalo area (see S2 Data). These studies both used CT scans, applied to a sample of the adult population. Since CT scans are known to miss a number of viable parenchymal lesions and a large share of extra-parenchymal lesions, but are effective at reliably identifying calcified NCC lesions, the parameter used for calibration is not total prevalence but the share of the adult population that have calcified lesions. The share of adults with calcified NCC lesions (with or without non-calcified lesions) was 18.8% in Rica Playa and 22.6% in Matapalo.

CystiHuman assumed that 20% (rounded value closest to the average of Rica Playa and Matapalo) could be used as a reasonable proxy for the average share of the adult population with calcified NCC lesions over the three villages used to calibrate CystiHuman (515, 566, 567).

# Share of all NCC cases that have one, two or more lesions

Seven community-level studies were identified that distinguish cases with single vs. multiple lesions and look at either the whole population or asymptomatic cases only [2-8]. Studies of the whole population and studies of asymptomatic cases yield almost the same results as most cases at community-level are asymptomatic. The studies are from Latin America, except two [4, 5] that are from India. There are 4 different communities/countries studied. Two successive studies were led by the same author (Prasad et al.) in the same community in India. Similarly, Del Brutto et al. led two successive studies in the same community in Ecuador [3, 6]. Meanwhile, in Mexico, Fleury et al. conducted two studies in different endemic communities in neighboring states [7, 8]. There were at most 4 years between successive studies in the same country or community, and we therefore assume that we can consider that multiple studies in the same community and even in geographically close communities in the same country can be considered as having sampled individuals from the same population. Results from studies within the same community (India, Ecuador) or country (Mexico) were therefore first pooled, then a random effects meta-analysis was applied to the results for the four different countries.

Using that method, the average share of individuals that have a single lesion is estimated at 72.5% [66.6-78.4%] (Fig A in S1 Text).

**Fig A: Proportion of all NCC cases that have a single lesion**

**
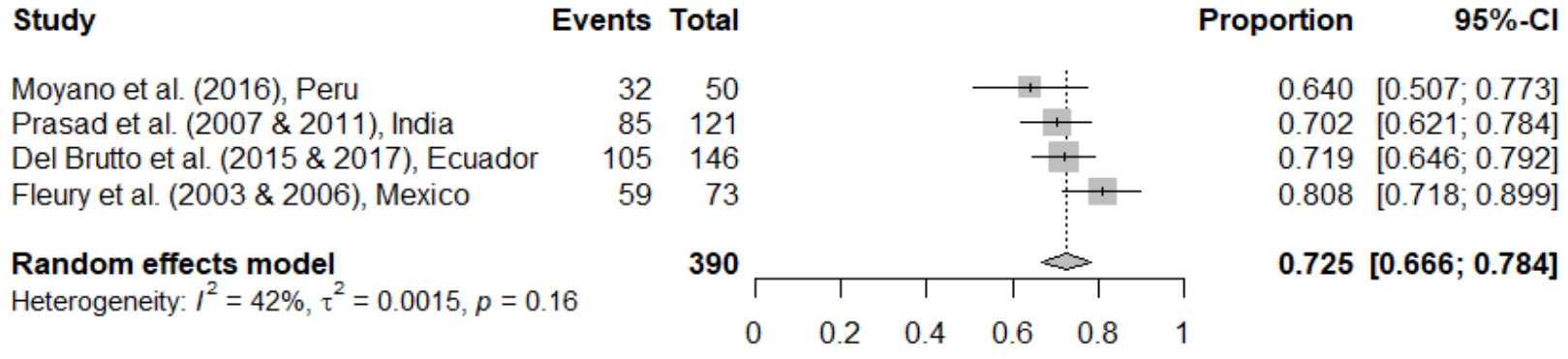
**

*Sources: [2-8]*

Using the same method for the share of NCC cases with exactly 2 lesions, pooling data from [2, 4-6], we find 16.9% [11.7-22.0%].

With a decreasing number of studies for higher numbers of lesions, we can only get a general sense of the rest of the distribution. Data on 3 and 4 lesions separately are generally not available, and one of the studies had a very small sample, meaning that there were no individuals with either 3 or 4 lesions. This leaves just one study [2] with non-zero numbers of individuals with 3 or 4 lesions, and it had 1 case with 3 lesions and 5 with 4 lesions. Meanwhile, the share of cases with 5 and more lesions, based on [2, 3, 6], is approximately 4% in the Ecuador studies with data and 10% in the Peru study. Table A in S1 Text summarizes the average values and 95% confidence intervals for the share of cases with one or two lesions.

**Table A: distribution of NCC cases per number of lesions**

| Indicator | Average | 95% CI: lower bound | 95% CI: upper bound |
| --- | --- | --- | --- |
| % of NCC cases with a single lesion | 72.5% | 66.6% | 78.4% |
| % of NCC cases with exactly 2 lesions | 16.9% | 11.7% | 22.0% |

# Stages of an NCC lesion

Both symptomatic and asymptomatic NCC lesions are important in the model. However, most available information comes from cases identified because they were symptomatic. The analysis below therefore focuses on the stages of a symptomatic NCC lesion. The model then assumes that asymptomatic lesions go through similar stages, with similar presentations on imaging, within the same timeframe, but without being associated with symptoms.

Maturation: new lesions begin with an asymptomatic maturation period, during which they are not visible on imaging. The maturation period has been estimated at 3 months (13 weeks) [9], which we assume to be the same for parenchymal and extra-parenchymal lesions, and for symptomatic and asymptomatic lesions.

Time to first symptoms: after a lesion has matured, it is visible on imaging but does not immediately cause symptoms. The distribution of timeframes from infection to first symptoms for symptomatic lesions was estimated based on a study of British soldiers infected in India [10]. There are multiple peaks in this distribution. We assume these peaks to reflect lesions in different locations. Indeed, the evolution of a lesion depends on whether it is parenchymal or extra-parenchymal: extra-parenchymal lesions are more likely to be associated with severe symptoms [11] and create symptoms at a later date than parenchymal lesions (in immigrants from endemic regions, symptoms often appear 10 years or more after the latest plausible date for infection [12]). We therefore assume that extra-parenchymal lesions drive the second peak in the distribution while parenchymal lesions drive the first peak. Based on this assumption, time from infection to first symptoms has been modelled as the sum of two Gamma distributions corresponding to each of the two possible locations:

$$Gamma \left( y,\alpha,\beta\right)= \frac{y^{\alpha-1}\beta^{\alpha}e^{-\beta y}}{\Gamma(\alpha)}$$

*y*: number of years from exposure to first symptoms; *α*: 2.94 for parenchymal lesions and 17.21 for extra-parenchymal lesions; *β*: 0.83 for parenchymal lesions and 1.10 for extra-parenchymal lesions.

Using these assumptions, the projected and observed distributions of timeframes from infection to first symptoms are very similar (Fig B in S1 Text). Further, the proposed model leads to an average time to first symptoms coherent with generally accepted timeframes for parenchymal lesions [9] and compatible with observations regarding extra-parenchymal lesions.

**Fig B: Incident cases as a function of the number of years between first symptoms and exposure, actual and projected**

*Source of ‘actual’ figures: [10]*

The duration *y* modelled through the gamma distribution above refers to the total period from infection to first symptoms, hence includes the maturation period. To obtain the duration from lesion maturity to first symptoms, this period should be removed (we use “Maximum” to ensure no duration is 0 or below):

$$lesion maturity to first symptoms = Maximum (y- 3 months, 1 week)$$

Time from first symptoms to death (disappearance or calcification) of an NCC lesion: most patients in endemic villages are untreated or solely treated for epilepsy. Hence, the model needs an estimate of the speed of calcification or disappearance of symptomatic parenchymal NCC lesions (viable or degenerating) in the absence of anthelminthic treatment. In practice, this generally means focusing on the placebo arm of existing studies.

A few studies [13-15] from India and Ecuador provide data on the likelihood of lesion death at multiple time points. These studies suggest (Fig C in S1 Text) that the evolution of the share of cysts that die over time, once first symptoms start, may be modelled by an exponential function. Assuming this is the case, data from studies that provide information on the share of cysts that have died at just one point in time can then be transformed into an estimated weekly death rate (Table B in S1 Text).

**Fig C: Share of active or transitional lesions that have died (calcified or disappeared), according to the time elapsed since first symptoms or diagnosis, in months**

*Source:[13-15]. The best fit curve corresponds to a death rate of 2.2% per week.*

**Table B: Weekly probability of death of an NCC lesion after the beginning of symptoms**

| Country & region | % that calcify or disappear, per week | % that calcify, of those that either calcify or disappear | Number that calcified or disappeared | Months of follow-up | % male | Age  (in years) | Imaging technique | Sample size* | Source |
| --- | --- | --- | --- | --- | --- | --- | --- | --- | --- |
| Ecuador | 2.4% | 7.9% | 63 | 24 in total | 53.0% | 40 ± 17.2 | CT & MRI | 63** | [13] |
| India | 7.7% | 22.2% | 18 | 7.08 | 65.0% | 24 ± 6 | CT | 20 | [16] |
| India, TN | 1.8% | 19.9% | 156 | 12 | 61.4% | 20.8 ± 12.8 | Thin slice CT | 210 | [14] |
| India, TN | NA | 18.4% | 185 | NA | 62.2% | 21 ± 12.2 | CT | 185 | [17] |
| India, DL | 2.3% | 50.0% | 104 | 10.8 ± 1.2 | 53.8% | 7.9 ± 2.8 | Plain & contrast CT | 104 | [18] |
| India, DL | 1.9% | 18.2% | 11 | 6 | 60.0% | 8.0 ± 2.6 | CT | 18*** | [19] |
| India, CH | 5.0% | 20.0% | 15 | 3 | 62.5% | 8.0 ± 2.3 | CT | 32 | [20] |
| India, CH | 2.7% | NA | NA | 3-6, median: 3.5 | 54.4% | 1.5 to 12.5 | CT | 86*** | [21] |
| India, UP | 3.7% | 33.3% | 21 | 6 | 58.8% | 16.0 ± 8.4 | CT | 34 | [22] |
| India, UP | 3.8% | 16.7% | 30 | 6 | 57.7% | 22 ± 14 | CT | 48 | [23] |

Codes for Indian States: TN: Tamil Nadu, DL: Delhi, CH: Chandigarh, UP; Uttar Pradesh

* Most studies were randomized controlled trials for anthelminthic drugs. As only the placebo arm of the trial is of interest to us, the ‘sample size’ correspond to the size of the placebo group.

** This is not a number of patients, but a number of cyst transitions within patients with multiple lesions.

*** This corresponds to patients in the placebo arm and with single lesions only.

The death rate used in the model is based on an unweighted average of data in Table B in S1 Text, including only studies with at least 40 cases within the sample of interest. The resulting average rate is 2.6% per week, with a plausible range of 1.8-3.8% (minimum and maximum for the studies included in Table B in S1 Text that have a sufficiently large sample). Table B in S1 Text is also useful for the analysis of the relative share of parenchymal lesions that calcify vs. disappear.

Note: one potential issue is that most studies in the table are from Asia rather than Latin America. At this stage, it is hard to tell whether results may be significantly different for the two regions, but based on experts’ opinions (H.G. & J.B.), the figures found in the table are generally coherent with what happens in Latin America.

# Probability of calcification vs. disappearance for a symptomatic parenchymal cyst

Lesions that die may disappear or leave a calcification. Table B and Fig D in S1 Text detail the share of lesions that calcify among those that have died. The average found for India studies [14, 16-20, 22, 23], using a random-effects model, is 25.2% [15.9-34.4%] (see Fig D in S1 Text). According to experts’ opinions (H.G. & J.B.), however, the behavior of cysts is somewhat different in Latin America, with a higher proportion of calcifications. For example, in one study [24] with data, 38% of cysts that calcified or disappeared in the placebo arm had calcified. However, only a minority of cysts had died at that point in the study, which may bias the results. After discussion, it has been estimated that the true figure for Latin America was likely lower than 38% and higher than the value for India, giving a plausible range of [25-38%] of cysts that will ultimately calcify vs. disappear. New field data, when available, may help refine this result.

**Fig D: Share of lesions that calcify among lesions that either calcify or disappear**
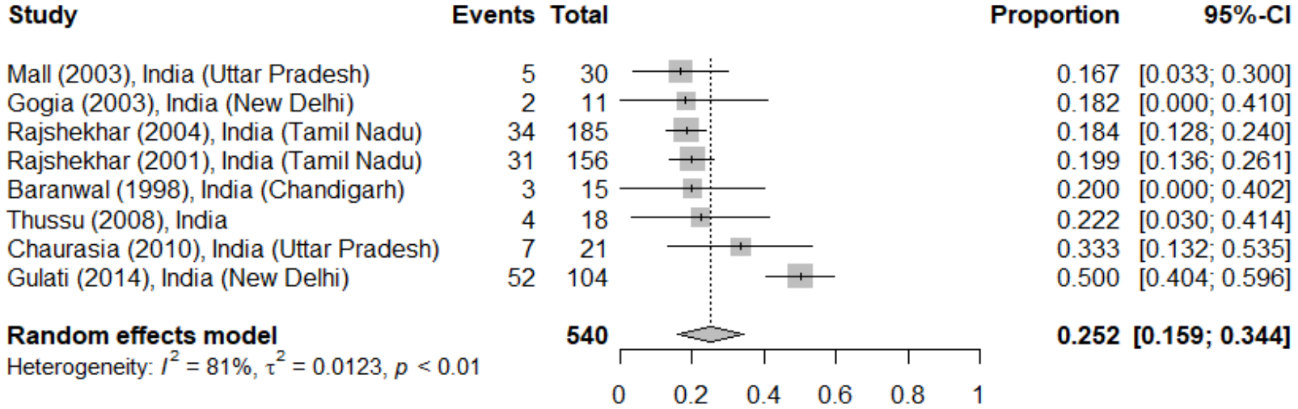


*Source: [14, 16-20, 22, 23]*

# Lifecycle of an extra-parenchymal NCC lesion

The lifecycle of an extra-parenchymal cyst after first symptoms appear is badly known, primarily because, when individuals arrive in hospital with the disease, they systematically receive treatment (medical or surgical). However, the “natural” lifecycle of an extra-parenchymal cyst is a relevant issue, as despite the severe symptoms and risk of death associated with those cysts, most individuals in endemic regions are not receiving care. Gathering more information on individuals with extra-parenchymal cysts at community level and how these have evolved (i.e., calcification or disappearance, but also whether these are associated with an easing of symptoms or whether any pre-existing hydrocephalus/ICH persists) will be essential.

# Adjustments to CystiAgent

Pray et al. [1] describe the CystiAgent model structure and parameters. Since this paper, adjustments have been made, some of which are of particular relevance to CystiHuman. These relate to: 1) demographic processes 2) the representation of eggs in the environment.

The original CystiAgent model [1] introduced short-term movement (e.g. business trips) in and out of endemic villages. This is particularly relevant when modelling interventions seeking to suppress transmission within villages, as it contributes to explain how parasite transmission may rebound after the end of the intervention through pathogen inflow. Neurocysticercosis levels, however, also depend on other demographic changes including births/deaths, and long-term movements in and out of the village (immigration and emigration). Those movements, and how they have been modelled, are described in S3 Text. They were integrated both in CystiAgent and CystiHuman.

The Pray et al. version of CystiAgent focuses on the number of eggs in the environment. In the revised CystiAgent, egg density is also computed. The density of eggs in the areas in which pigs spend time (mostly close to their owner’s home, and to a certain extent in a larger area in the village) is a driver of parasite transmission. Meanwhile, the overall density of eggs in the village is used as an input to CystiHuman, and drives environmental contamination risk. Transmission models providing solely egg numbers as an output could however be used to apply CystiHuman by translating numbers into a density using total village area.

# References

1. Pray IW, Wakeland W, Pan W, Lambert WE, Garcia HH, Gonzalez AE, et al. Understanding transmission and control of the pork tapeworm with CystiAgent: a spatially explicit agent-based model. Parasites & vectors. 2020;13(1):1-13.

2. Moyano LM, O'Neal SE, Ayvar V, Gonzalvez G, Gamboa R, Vilchez P, et al. High Prevalence of Asymptomatic Neurocysticercosis in an Endemic Rural Community in Peru. PLoS neglected tropical diseases. 2016;10(12):e0005130.

3. Del Brutto OH, Arroyo G, Del Brutto VJ, Zambrano M, García HH. On the relationship between calcified neurocysticercosis and epilepsy in an endemic village: A large‐scale, computed tomography–based population study in rural Ecuador. Epilepsia. 2017;58(11):1955-61.

4. Prasad KN, Verma A, Srivastava S, Gupta RK, Pandey CM, Paliwal VK. An epidemiological study of asymptomatic neurocysticercosis in a pig farming community in northern India. Transactions of the Royal Society of Tropical Medicine and Hygiene. 2011;105(9):531-6.

5. Prasad A, Gupta RK, Pradhan S, Tripathi M, Pandey CM, Prasad KN. What triggers seizures in neurocysticercosis? A MRI-based study in pig farming community from a district of North India. Parasitology International. 2007;57(2):166-71.

6. Del Brutto OH, Salgado P, Lama J, Del Brutto VJ, Campos X, Zambrano M, et al. Calcified neurocysticercosis associates with hippocampal atrophy: a population-based study. The American journal of tropical medicine and hygiene. 2015;92(1):64-8.

7. Fleury A, Morales J, Bobes RJ, Dumas M, Yánez O, Piña J, et al. An epidemiological study of familial neurocysticercosis in an endemic Mexican community. Transactions of the Royal Society of Tropical Medicine and Hygiene. 2006;100(6):551-8.

8. Fleury A, Gomez T, Alvarez I, Meza D, Huerta M, Chavarria A, et al. High Prevalence of Calcified Silent Neurocysticercosis in a Rural Village of Mexico. Neuroepidemiology. 2003;22(2):139-45.

9. Garcia HH, Gonzalez AE, Gilman RH, Cysticerosis Working Group in P. Diagnosis, treatment and control of Taenia solium cysticercosis. Current Opinion in Infectious Diseases. 2003;16(5):411-9.

10. Dixon HBF, Lipscomb FM. Cysticercosis : an analysis and follow-up of 450 cases. London: H.M. Stationery Office; 1961.

11. García HH, Gonzalez AE, Evans CAW, Gilman RH, Cysticercosis Working Group in P. Taenia solium cysticercosis. The Lancet. 2003;362(9383):547-56.

12. Nash TE, O'Connell EM, Hammoud DA, Wetzler L, Ware JM, Mahanty S. Natural History of Treated Subarachnoid Neurocysticercosis. The American journal of tropical medicine and hygiene. 2020;102(1):78-89.

13. Montgomery MA, Ramos M, Kelvin EA, Carpio A, Jaramillo A, Hauser WA, et al. A longitudinal analysis of albendazole treatment effect on neurocysticercosis cyst evolution using multistate models. Transactions of the Royal Society of Tropical Medicine and Hygiene 2019;113(12):781.

14. Rajshekhar V. Rate of spontaneous resolution of a solitary cysticercus granuloma in patients with seizures. Neurology. 2001;57(12):2315-7.

15. de Souza A, Nalini A, Kovoor JME, Yeshraj G, Siddalingaiah HS, Thennarasu K. Natural history of solitary cerebral cysticercosis on serial magnetic resonance imaging and the effect of albendazole therapy on its evolution. Journal of the Neurological Sciences. 2009;288(1):135-41.

16. Thussu A, Chattopadhyay A, Sawhney IMS, Khandelwal N. Albendazole therapy for single small enhancing CT lesions (SSECTL) in the brain in epilepsy. Journal of Neurology, Neurosurgery & Psychiatry. 2008;79(3):272-5.

17. Rajshekhar V, Jeyaseelan L. Seizure outcome in patients with a solitary cerebral cysticercus granuloma. Neurology. 2004;62(12):2236-40.

18. Gulati S, Jain P, Sachan D, Chakrabarty B, Kumar A, Pandey RM, et al. Seizure and radiological outcomes in children with solitary cysticercous granulomas with and without albendazole therapy: A retrospective case record analysis. Epilepsy Research. 2014;108(7):1212-20.

19. Gogia S, Talukdar B, Choudhury V, Singh Arora B. Neurocysticercosis in children: clinical findings and response to albendazole therapy in a randomized, double-blind, placebo-controlled trial in newly diagnosed cases. Transactions of the Royal Society of Tropical Medicine and Hygiene. 2003;97(4):416-21.

20. Baranwal AK, Singhi PD, Khandelwal N, Singhi SC. Albendazole therapy in children with focal seizures and single small enhancing computerized tomographic lesions: a randomized, placebo-controlled, double blind trial. The Pediatric Infectious Disease Journal. 1998;17(8):696-700.

21. Singhi P, Ray M, Singhi S, Khandelwal N. Clinical Spectrum of 500 Children With Neurocysticercosis and Response to Albendazole Therapy. Journal of Child Neurology. 2000;15(4):207-13.

22. Chaurasia RN, Garg RK, Agarwall A, Kohli N, Verma R, Singh MK, et al. Three day albendazole therapy in patients with a solitary cysticercus granuloma: a randomized double blind placebo controlled study. The Southeast Asian journal of tropical medicine and public health. 2010;41(3):517.

23. Mall RK, Agarwal A, Garg RK, Kar AM, Shukla R. Short Course of Prednisolone in Indian Patients with Solitary Cysticercus Granuloma and New‐onset Seizures. Epilepsia. 2003;44(11):1397-401.

24. Garcia HH, Pretell EJ, Gilman RH, Martinez SM, Moulton LH, Del Brutto OH, et al. A Trial of Antiparasitic Treatment to Reduce the Rate of Seizures Due to Cerebral Cysticercosis. The New England Journal of Medicine. 2004;350(3):249-58.
