# Supplementary material for: CystiHuman: A model of human neurocysticercosis
Source: PLoS Comput Biol. 2022 May 19;18(5):e1010118. doi: 10.1371/journal.pcbi.1010118 (PMC9159625; doi:10.1371/journal.pcbi.1010118)
Supplement: S2 Text — Table A in S2 Text: CT scan assessment (NCC or not) and cause of epilepsy (NCC-related or not) at diagnosis. Table B in S2 Text: proportion of cases of ICH/hydrocephalus that have extra-parenchymal lesions, by country. Table C in S2 Text: Delay between first symptoms and treatment for patients with ICH or hydrocephalus that ultimately sought treatment. Table D in S2 Text: Number of surgeries per surgical NCC case & share of surgical NCC cases that are cured. Fig A in S2 Text: Share of individuals with a single lesion among symptomatic NCC cases. (DOCX) [file pcbi.1010118.s002.docx]

Supporting information 2 – NCC symptoms (excluding deaths)

This section details the data used to model NCC-related symptoms (epilepsy/seizures, intracranial hypertension – or ICH – and hydrocephalus), their timing and associated costs, excluding deaths, births and migration, and data already described in S 1 Text. This includes:

1. The probability of lifetime epilepsy and seizure recurrence among individuals with NCC.
2. The share of individuals with epilepsy that have multiple lesions
3. The probability of ICH/hydrocephalus and its association with extra-parenchymal vs. parenchymal lesions.
4. The likelihood, timing and type of treatment for active epilepsy or ICH/hydrocephalus.

# Probability of epileptic seizures and lifetime epilepsy

## Added risk of lifetime epilepsy for individuals with NCC

Few studies provide information allowing for a direct comparison of individuals with and without NCC and their epilepsy risk. For example, [1] found 9/109 (8.3%) individuals with epilepsy among NCC cases in Atahualpa, Ecuador, and 30/1110 (2.7%) among non-NCC cases. The difference in epilepsy rates between NCC cases and non-NCC cases in this context is 5.6% [1.5-12.3%] – a very large confidence interval.

Meanwhile, information on the odds of having NCC when one has epilepsy vs. when one does not is more common: [2], in a meta-analysis of studies in Latin America found an odds ratio (OR) of 2.8 [1.9-4.0] ([3] found an odds ratio of 2.2 [0.9-3.5] (3.25 [2.13-4.93] for the Americas as a whole, but for modelling in Peru, we chose to use data from Latin America specifically). Furthermore, the median proportion of individuals with neurocysticercosis among people with epilepsy was *% NCC among people with epi* = 32.3% [26.0-39.0%], while lifetime prevalence of epilepsy was *LTE* = 15.8/1000 [13.5-18.3] for Latin America [2]. Overall NCC prevalence is badly known. However, [4] reviews a number of studies on various communities across Latin America and finds values from 4 to 24% with a median at 10.4%.

We use this information to derive an estimate of the added risk for lifetime epilepsy associated with neurocysticercosis, as below:

$$\frac{NCC associated epi risk}{(1- NCC associated epi risk)}=(OR-1)*LTE* \frac{1-\% NCC among people with epi}{1- overall NCC prevalence}$$

This leads to an added risk of lifetime epilepsy associated with neurocysticercosis of 2.10% [1.05-3.69%]. The total risk would be 3.3%. Using [3] instead, we would find an added risk of 2.6% and total risk of 3.8%.

## Recurrence of seizures after the disappearance of a parenchymal NCC lesion

When cysts disappear entirely, symptoms sometimes recur. [5] suggests that 11.9% [7.2-18.2%] have at least one new episode, while [6] found this was the case for 7.6% of the children in the study. Based on that, the model assumes that seizure recurrence happens in around 1/10 cases. The model further assumes that these seizures may be modelled as happening almost immediately after the disappearance of the lesion.

## Seizure recurrence for NCC cases with parenchymal lesions

A center providing free epilepsy diagnosis and treatment opened in 2007 in the Tumbes region (North-West) of Peru. Patients were originally identified through systematic community outreach, though as the center became better known, visitors were also attracted through word of mouth. Overall, however, the patients represent a relatively unbiased reflection of epilepsy cases in the community. Data from all baseline visits were recently digitalized, and represent an extensive source of information. For each individual, the database generally provides their demographics, epilepsy status and origin, treatment, the timing of the latest seizure and duration of the disease, and the result of a CT scan, including the nature, location and stage of lesions (see S1 Data for data). Follow-up visits have yet to be digitalized but will provide further information.

This dataset was used to estimate three key figures (model observables and parameters): 1) the share of calcified NCC cases with epilepsy that have active epilepsy, 2) the share of NCC cases with active epilepsy that have non-calcified lesions, and 3) the frequency of seizures associated with non-calcified NCC lesions.

The figures were computed for cases with solely parenchymal lesions and no stated treatment likely to affect the result (i.e., no anti-epileptic treatment for 1) and 3) as it may contribute to stop the seizures, and no anthelminthic treatment for 2) as it may affect the lesion calcification/disappearance process).

Note: 53% of cases were coded with “no answer”. This is the same for most questions of the clinical questionnaire. These “no answers” likely seldom reflect individuals that did not have a clinical interview, as the large majority of them (98%) answered at least some of the clinical questions. A suspicion is that many of these “no answer” correspond to cases in which the space was left empty because there was no specific treatment to report rather than because there was no answer. We therefore chose to only exclude cases with answers explicitly mentioning treatment.

Cases were identified based on the CT scan results and the assessment of the origin of epilepsy. We divided CT scan results into three groups: no NCC (normal CT or CT showing anomalies stemming from issues other than NCC), certain or likely NCC, and compatible with NCC (CT scans showing anomalies such as calcified granulomas that could be NCC but that the radiologist did not explicitly relate to NCC). Epilepsy assessments were also divided into three categories: 1) those that explicitly linked the epilepsy symptoms to NCC, 2) those that explicitly linked the epilepsy symptoms to other causes (such as idiopathic epilepsy, head trauma, etc.), and 3) those whose etiology was deemed unclear. For the purpose of computing statistics, we focused on individuals with CT scans showing certain/likely NCC or anomalies compatible with NCC and having a diagnosis of epilepsy explicitly linked to NCC. Results are provided in S2 Table 1.

**Table A: CT scan assessment (NCC or not) and cause of epilepsy (NCC-related or not) at diagnosis**

|  | **CT scan diagnosis: no NCC** | **CT scan diagnosis: certain/likely NCC** | **CT scan diagnosis compatible with NCC** |
| --- | --- | --- | --- |
| % with NCC as a cause of epilepsy | 5.9% (40/675) | 58.8% (104/177) | 40.4% (120/297) |
| % with a non-NCC cause of epilepsy | 64.4% (435/675) | 14.7% (26/177) | 24.2% (72/297) |
| % with an unclear cause of epilepsy | 29.6% (200/675) | 26.6% (47/177) | 35.4% (105/297) |
| % with NCC as a cause among cases with known epilepsy causes | 8.4% (40/475) | 80.0% (104/130) | 62.5% (120/192) |

Table A in S2 Text shows that a majority of epilepsy cases with an NCC diagnosis (and likely of cases with a diagnosis compatible with NCC) had epilepsy caused by NCC. Meanwhile, a small number of cases with a clean CT scan nevertheless had a diagnosis of epilepsy caused by NCC. This may be due to error or possibly, in some cases, to past NCC lesions that were associated with seizures then disappeared without leaving a calcification.

The majority of NCC cases in Table A in S2 Text were untreated parenchymal NCC cases. These were used to compute the observables below.

*Share of never treated calcified parenchymal NCC cases with epilepsy that have (long-term) active epilepsy*

This indicator helps distinguish between cases with long-term active disease and a high burden and cases that had seizures that rapidly subsided. “Long-term” active epilepsy is defined as a case of active epilepsy in which the first seizure happened at least 7.2 years ago. Indeed, we have decided to consider only lesions that calcified at least 5 years ago (otherwise cases that had seizures up to calcification could be included in the active epilepsy figures). For lesions that were associated with seizures prior to their death, 95% are expected to have calcified or disappeared within 2.2 years (based on the 2.6% weekly death rate estimated in S1 Text), hence we chose to focus only on NCC cases whose first seizure was at least 2.2 + 5 = 7.2 years ago. There are 68 such epilepsy cases, 45 of whom (or 66%) have active epilepsy.

*Share of never treated active epilepsy parenchymal NCC cases that have non-calcified lesions*

In the dataset, 138 individuals had active epilepsy and solely parenchymal lesions identified as likely or surely related to NCC. Among these, 22, or 15.9% (95% CI 10.3-23.1%) had at least one non-calcified lesion.

*Frequency of seizures associated with parenchymal NCC lesions (in never treated cases with active epilepsy)*

The dataset does not, strictly speaking, provide the frequency of seizures associated with a single lesion, but information enabling the computation of seizure frequency in individual NCC patients. However, only a small share of all NCC lesions leads to seizures hence seizures in patients with multiple lesions are generally driven by only one of these lesions. The frequency of seizures in NCC patients is therefore a good approximation of the frequency of seizures associated with one lesion.

Meanwhile, the dataset does not provide the date of each seizure but the estimated date of the latest seizure. We use the time span between the latest seizure and patient assessment to estimate the duration between seizures. This assumes there is no bias in the choice of the date of visit. This bias should be minimal in patients who were selected through community outreach, assumed to form the majority of the sample.

There are 67 never treated cases in the dataset with solely parenchymal calcified lesions and active epilepsy caused (according to the physician’s assessment) by NCC. The median time since the latest seizure is 12.6 weeks. This corresponds to an estimated seizure frequency of 0.05/week. There is a large uncertainty on this figure, however, the economic and social burden of NCC will depend largely on the number of cases with active vs. inactive epilepsy rather than on an exact reflection of seizure frequency.

*Probability for anti-epileptic treatment to be successful for NCC cases at the calcified stage*

Regarding the share of individuals with “successful” or “unsuccessful” treatment, the general practice in Peru seems to be to discontinue treatment after 2 years without seizures. In India, [7, 8] found that 24% and 25% of cases with calcified cysts and antiepileptic treatment had seizure recurrence at one year, while [9] found that 20% of the study sample (or 12 individuals) had seizure recurrence at 2 years. For Latin America, [10] studied a group of 210 epileptic calcified NCC cases treated with anti-epileptic drugs. Total follow-up was 3 years and 49.1% (103/210) of the cohort had a subsequent seizure. A time-to-event analysis showed that only 36.0% remain free of epilepsy over the whole 3-year period while this number is 46.7% [38.6%-54.4%] over a two-year period. These latter figures were therefore used to define the rates of “successful” vs. “unsuccessful” treatment. Strictly speaking, these refer to NCC cases with epilepsy and one or several calcified lesions rather than what happens for a single NCC lesion. However, in most cases, even in individuals with several calcified lesions, only one lesion is responsible for the seizures [10] – the 46.7% figure is therefore used to describe success in making an individual calcified lesion quiescent.

*Likelihood of seizure relapse after treatment*

Studies have suggested that the likelihood of further seizures after AED treatment stops, for NCC cases with a history of seizures at the calcified stage, is high (25/30 cases or 83%) [11]. However, it also seems that most seizures are concentrated in individuals that had breakthrough seizure during treatment. In [10], only 2/31 individuals that had been treated and seizure free for at least 2 years had further seizures, while a history of breakthrough seizures increased the risk of further seizures by a factor 9 as compared to successful treatment. The model simplifies these results by assuming that, after 2 years with successful treatment, NCC cases no longer have seizures, while, if treatment was unsuccessful, seizure frequency after the end of treatment is similar to seizure frequency in untreated cases.

# Share of individuals with epilepsy that have multiple lesions

We reviewed community studies providing information on NCC cases with epilepsy and their number of lesions. We excluded studies focusing either solely on a specific type of lesion (e.g., calcified lesions only) and studies with fewer than 10 cases, and grouped successive studies by the same authors within the same community. We found 6 studies in 5 distinct communities. Using a random effects meta-analysis, we found a significant variability in the proportion of individuals with a single lesion, with an average of 47% [34-61%]. There is one significant outlier: a study by Singh et al. [12] analyzing the association between epilepsy and toxocariasis and cysticercosis in a community endemic for both. Should that community be excluded, the average would become 42% [32-53%], with a heterogeneity of 42% and p = 0.16.

**Fig A: Share of individuals with a single lesion among symptomatic NCC cases**


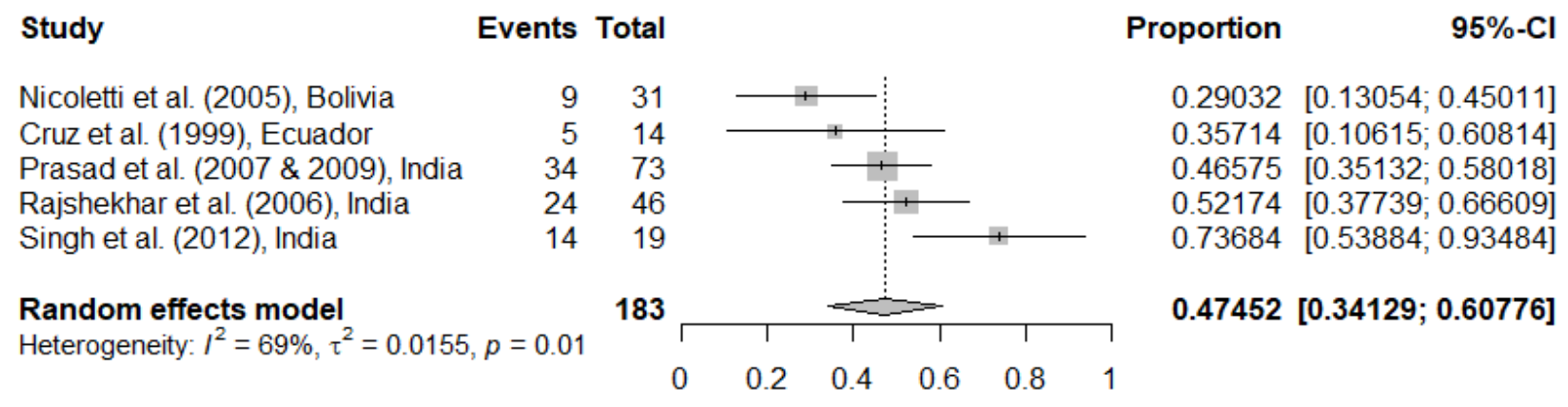


Source: [12-17]

The average found through this meta-analysis is significantly lower than the proportion of NCC cases with a single lesion in community-based studies of asymptomatic cases or the whole population: 72.5% [67-78%]. This suggests that, in community level studies, NCC cases with multiple lesions are significantly more likely (about 3 times more, using the 72.5% and 47% figures) to be diagnosed as having epilepsy than NCC cases with single lesions. This result drove the decision to associate epilepsy risk to individual lesions then combine these risks to model host symptomatology, rather than attribute a flat, unique risk to all NCC cases, irrespective of the number of lesions they harbor.

# Likelihood of ICH/hydrocephalus

The likelihood of developing an extra-parenchymal lesion is given by the tuning parameter *ξ*. Extra-parenchymal lesions were only modelled if they lead to ICH or hydrocephalus. Meanwhile, some parenchymal lesions can also lead to ICH/hydrocephalus. Tuning *ξ* can be done using several methods, all imprecise. One is to estimate the prevalence of cases with extra-parenchymal lesions (assuming the large majority of those will lead to symptoms). Another, which can be used as a second check of model data, is estimate the number of ICH or hydrocephalus cases as compared to epilepsy cases. A third consists in comparing recorded epilepsy deaths and recorded ICH/hydrocephalus deaths among NCC cases. Once *ξ* is tuned, knowledge of the relative weight of parenchymal NCC cases among hydrocephalus/ICH cases is sufficient to tune *π_i_.*

## Prevalence of NCC cases with extra-parenchymal lesions

The prevalence of NCC cases with extra-parenchymal lesions in clinical settings is relatively well-known, but in community-level surveys, the tools most often used to assess NCC prevalence (CT-scans) are not well-adapted to detect such lesions. To estimate a minimum prevalence for cases with extra-parenchymal lesions (solely or alongside parenchymal lesions), symptomatic or not, in the population of endemic regions in Peru, we use the results of community-level surveys that proposed MRIs to at least part of the population.

[18] took blood samples from 514 consenting individuals over 2 years old in an endemic community of Northern Peru, in the Piura region. Those strongly positive on ELISA were offered an MRI with 11 out of 12 accepting. 5 of those, corresponding to 1% of the target population, had extra-parenchymal lesions.

Another survey by the Cysticercosis Working Group in Peru (S. O’Neal, personal communication, January 12, 2021), looked into urine antigen levels in the population of endemic villages in the region of Tumbes, in Northern Peru, and gave MRIs to all consenting individuals with high antigen levels (Ag ODR ≥3). During the pilot, out of 978 eligible individuals 12 years old or above that provided a urine sample, 17 had high antigen level, 13 underwent an MRI, and 8 had extra-parenchymal subarachnoid neurocysticercosis (none had intraventricular disease), corresponding again to 1% of the target population. The main study took urine samples from 8315 eligible individuals over 2 years old in multiple villages from the region of Tumbes, in Northern Peru, and found 81 with high antigen level. 63 underwent an MRI, and 18 of them had extra-parenchymal lesions (17 subarachnoid disease, and 1 ventricular NCC), corresponding to close to 0.3% of the target population.

Given that these methods only identify part of existing extra-parenchymal lesions, we use 0.3% as a lower boundary for the prevalence of extra-parenchymal lesions in endemic communities in Peru. We also used a 5% upper boundary for that prevalence, based on experts’ opinions (S.E.O).

## Intracranial hypertension (ICH) and/or hydrocephalus among clinical patients with solely parenchymal lesions

*Active/transitional parenchymal NCC among ICH/hydrocephalus cases:*

Five studies with data were found (Table B in S2 Text). The overall expected share of ICH/hydrocephalus cases that happen in individuals with extra-parenchymal lesions is 91.4% [82.7-100%], hence our choice to use 8.6% as the share, among NCC cases with ICH/hydrocephalus, of individuals with solely parenchymal lesions. Note that many of these cases may have transient ICH/hydrocephalus as ICH/hydrocephalus is generally not found among individuals with calcified NCC (see below).

**Table B: proportion of cases of ICH/hydrocephalus that have extra-parenchymal lesions, by country**

| Country | ICH or hydrocephalus among cases with extra-parenchymal lesions | Number of cases of ICH or hydrocephalus | % of ICH/hydrocephalus cases that have extra-parenchymal lesions | Source |
| --- | --- | --- | --- | --- |
| Ecuador | 68 | 92 | 74% | [19] |
| Portugal | 12 | 13 | 92% | [20] |
| Mexico | 154 | 166 | 93% | [21] |
| USA | 28 | 28 | 100% | [22] |
| USA | 6 | 6 | 100% | [23] |

*ICH/hydrocephalus and calcified NCC:*

[19, 24, 25] provide information on intracranial hypertension (ICH) cases among individuals with calcified NCC. Among a total of 252 cases of calcified NCC in these 3 studies, none had ICH. Meanwhile, [22-24] provide information on hydrocephalus cases among individuals with calcified NCC. Among a total of 150 cases of calcified NCC in these 3 studies, 4 (2.7%) had hydrocephalus. This suggests that ICH and hydrocephalus are at most very rare in calcified NCC, and the model simplifies the situation by assuming that calcified NCC is not associated with either.

# Treatment likelihood, delay, and type

## Treatment gap for NCC patients with epilepsy/seizures and ICH/hydrocephalus

Not every person with NCC gets optimally treated. NCC cases with epilepsy that take antiepileptic drugs often use subtherapeutic doses that are insufficient to control the disease. The model only counts as “treated” those individuals taking a treatment that is appropriate to their condition. In the Tumbes dataset (see S1 Data), 437 out of 1570 active epilepsy patients (28%) had ever been treated with anti-epileptic drugs (AEDs). Out of those, considering only those for whom timing/regularly information is available, 8% (33/434) have stopped taking AEDs or are taking them irregularly. Therefore, an estimated 25.7% of active epilepsy patients are taking AEDs regularly. This aligns with a 2018 survey in Northern Peru [26] that also found a 75% treatment gap for people with epilepsy. This value was therefore used for the rural endemic communities of North-West Peru targeted in this model.

Note: the 25% figure is obtained if individuals with blank answers (officially coded as “no answer”) are included, in line with the choice in Section 1.3 of this document. If only individuals with clear answers are included, then the treatment gap would go down to 42%, a figure likely too optimistic given the context of these highly endemic communities.

With ICH/hydrocephalus, very little is known of treatment likelihood. The disease is more severe than epilepsy, but may start in a less recognizable manner, leading to important treatment delays among those that seek treatment (see Section 2.2 below for an estimate of those delays). Further, ICH/hydrocephalus should be treated in a tertiary care setting, making treatment less accessible than for epilepsy. In practice, it is expected that very few individuals will have sought treatment in endemic communities in North-West Peru. No estimate is available. In Peru, a small cohort of nine individuals were diagnosed with extra-parenchymal (subarachnoid) NCC through community outreach in endemic villages. All were referred to a specialist. At check-up 18 months afterward, though they all had symptoms, none had not seen a specialist (S. O’Neal, personal communication, July 16, 2021). Meanwhile, in the Tumbes dataset (S1 Data), 67 patients with epilepsy and a CT scan had signs of enlargement of the extra-parenchymal spaces and/or successful CSF derivation, and 4 of them (6%) have a record of surgical treatment. Based on this limited information and discussion with experts, it was decided that the treatment gap in highly endemic villages in North-West Peru was likely higher than 90%, with 95% being selected for model projections.

## Treatment delay for NCC patients with ICH or hydrocephalus

For NCC patients that are treated for ICH/hydrocephalus, there are often delays between first symptoms and treatment. Studies [27-31] provide some information in this regard. There may have been a decline in treatment delay over time, but there is too little information to tell. Data is sometimes ambiguous as the beginning of symptoms can be subtle, and some early symptoms, in cases with both epilepsy and ICH/hydrocephalus at diagnosis, may have been related to epilepsy rather than to ICH. Further, beyond [27], other studies have extremely small samples. To check whether [27] may be an outlier, we compared the estimated distribution of cases in [27] with that found by pooling all other studies taken together. These distributions were coherent, which is reassuring. Pooling all five studies, we find the following distribution: 37% of cases delay treatment by less than 1 month, 36% by 1-6 months, 10% by 6-12 months, and 19% by over a year. For cases that delay treatment by over a year, we assume that, in the vast majority of cases, treatment is not delayed for more than 6 years (maximum for all studies excluding [27]).

Precision on this distribution is not essential to the model, and the estimates serve primarily to avoid concentrating all projected ICH/hydrocephalus-related hospitalization and surgery costs on the day symptoms begin.

**Table C: Delay between first symptoms and treatment for patients with ICH or hydrocephalus that ultimately sought treatment**

| location & date | average | median | range | number | 1 month or less | >1 & up to 6 months | >6 & up to 12 months | > 12 months | source |
| --- | --- | --- | --- | --- | --- | --- | --- | --- | --- |
| Spain, 1981 | 10.9 to 22.6 months* | 5 to 9 months* | 3 days -72 months | 11 | 2 or 0* | 6 or 5* | 2 | 1 or 4* | [31] |
| USA, 1992 | 15.75 months | 10.5 months | 6-36 months | 4 | 0 | 0 | 2 | 2 | [30] |
| Brazil, 2002 | NA | 2.67 months | 0-17 years | 160 | 61 | 57 | 13 | 29 | [27] |
| USA, 2011 | 35 days | 5 days | 1-180 days | 11 | At least 6 | 5 or fewer | 0 | 0 | [28] |
| India, 2019 | 11.4 months | 3 months | 3-36 months | 5 | 0 | 3 | 1 | 1 | [29] |

* Figures vary according to whether subtle or plausible early signs are included

## Probability, frequency and timing of surgical treatment for ICH or hydrocephalus

ICH and hydrocephalus are serious, potentially deadly symptomatic presentations which generally require brain surgery. Surgeries include, in particular, cyst excision and diversion of cerebrospinal fluid. Multiple surgeries may be needed, and shunts on NCC patients may need revisions or replacement. However, a few cases with ICH or hydrocephalus (often milder cases) appear to be treated successfully through medical treatment (anti-helminthic drugs) without surgery. This corresponds to a minority of cases. In [28], 2 of 7 cases with hydrocephalus and NCC were not treated with surgery. In [20], this was the case of 2 out of 13 cases with ICH and/or hydrocephalus, while in [32], 4 or 19 cases with subarachnoid cysts and hydrocephalus were treated medically. Overall, the model will assume that approximately 20 % [7-32%] of cases of ICH or hydrocephalus do not require surgical treatment.

Brain surgeries are very costly, so modelling the number of surgeries is very important. This number obviously depends on the number of individuals having surgical treatment. However, this may lead to an underestimate of costs as many individuals have multiple surgeries, for example, shunt placement followed by multiple shunt replacements. Estimating the number of surgeries per surgical case is not straightforward.

We considered studies providing information on surgeries, reoperations and/or shunt revisions for at least 20 surgical NCC cases [27, 31, 33-39]. Across these studies, the average number of surgeries/reoperations/shunt revisions per surgical NCC case ranged from 1.0 to 2.3 per case (weighted and unweighted averages: 1.8 and 1.7 respectively). These studies, however, focused on diverse populations, sometimes specific surgical treatment, and provide varying (sometimes incomplete) levels of information. Some detail surgeries and re-operations, including a significant number of shunt placements, but there is no mention of shunt revisions (despite these being very common in all studies with data). In other cases, it is unclear how successive surgeries for the same patient were counted. Despite these limitations, Table D in S2 Text shows two things 1) multiple surgeries (including shunt revisions) are common 2) endoscopic treatment appears to be associated with fewer surgical procedures than other procedures, in particular shunting.

**Table D: Number of surgeries per surgical NCC case & share of surgical NCC cases that are cured***

| Location and date | Surgical cases | Focus (type of patients / type of treatment) | Total surgeries, including shunting (number of shunt procedures in brackets) | Cured cases | Ratio (surgeries / cases) | % of cured cases | Source |
| --- | --- | --- | --- | --- | --- | --- | --- |
| Spain, 1981 | 10 | Hydrocephalus | 21 (8) | 2 or 3 | 2.1 | 20-30% | [31] |
| Brazil, 1986 | 69 | ICH | 98 (56) | ≤ 5 | 1.4 | ≤ 7% | [33] |
| Mexico, 1987 | 92 | Arachnoiditis | 258 (0) | 0 | 1.7 | 0% | [34] |
| Brazil, 2002 | 160 | Any | 339 (59) | NA | 2.1 | NA | [27] |
| USA, 2002 | 21 | Hydrocephalus cases requiring shunt treatment | 49 (23) | 0** | 2.3 | 0%** | [35] |
| India, 2007 | 22 | Intraventricular NCC, endoscopy | 22 (0) | 22** | 1.0 | 100%** | [36] |
| India, 2007 | 21 | Intraventricular NCC, endoscopy | 21 (0) | 0** | 1.0 | 0%** | [37] |
| Mexico, 2009 | 115 | Intraventricular & subarachnoid, half traditional treatment, half endoscopy | 232 (175) | 42** | 2.0 | 37%** | [38] |
| USA, 2009 | 31 | Any | 41 (16) | 13 | 1.3 | 42% | [39] |

** Including cases that underwent multiple surgical interventions, provided that the last intervention led to long-term recovery. On the other hand, individuals needing permanent brain shunts (or other medical devices) were not considered “cured”.*

*** The sample was not representative (focus on shunts and shunt malfunction in [35], focus on patients treated with endoscopic treatment in [36] [37] and comparison of an equal number of endoscopic and traditional treatments in [38].*

Discussions with experts (H.G. & J.B.) from Peru help complement the picture. They suggest the current number of surgical procedures per surgical case in Peru is low: around 1.0-1.3 per surgical case. Hence, for the model, we used a value of 1.2 and a plausible range of [1.0-2.3] for the number of surgeries per surgical case. This value should be refined as more data are collected.

Even more limited data is available on the timing of re-operations and shunt revisions. [33] states that 67 of 88 reoperations (76.1%) took place in the first year, and 10 (11.4%) during the second year, while [35] found that 78.3% (18) of 23 shunt failures occurred within the first year, while 95.7% (22) occurred within the first 3 years. Pooling this information, we may estimate that 77.2% of all re-operations/shunt revisions take place during the first year, 11.4% during the second year, 7.1% during the third year, and 4.3% during the subsequent years. This is a possible avenue to improve the precision of the model. So far, however, once surgical treatment starts, all surgeries are modelled as taking place at the same time.

Finally, the long-term outlook of NCC cases with ICH/hydrocephalus is unclear. By definition, people solely undergoing medical treatment are expected to be cured of ICH/hydrocephalus (as a rule, when symptoms persist, other medical procedures are undertaken). Among people who did not get treated and survived, the long-term outlook is unknown. Finally, among people who were treated surgically, some are “cured”, typically through complete cyst removal. Data suggests that “cured” cases may represent an increasing share of all cases (as improved procedures become more common), and that they represent less than half of all cases. Other cases will spend the rest of their life with a medical device, typically a shunt. Overall, long-term functioning of NCC cases that survive ICH/hydrocephalus varies a lot, but a significant proportion of both “cured” individuals and people with long-term shunting may be able to go back to work and resume normal activities.

# References

1. Del Brutto OH, Arroyo G, Del Brutto VJ, Zambrano M, García HH. On the relationship between calcified neurocysticercosis and epilepsy in an endemic village: A large‐scale, computed tomography–based population study in rural Ecuador. Epilepsia. 2017;58(11):1955-61.

2. Bruno E, Bartoloni A, Zammarchi L, Strohmeyer M, Bartalesi F, Bustos JA, et al. Epilepsy and neurocysticercosis in Latin America: a systematic review and meta-analysis. PLoS neglected tropical diseases. 2013;7(10):e2480.

3. Debacq G, Moyano LM, Garcia HH, Boumediene F, Marin B, Ngoungou EB, et al. Systematic review and meta-analysis estimating association of cysticercosis and neurocysticercosis with epilepsy. PLoS neglected tropical diseases. 2017;11(3):e0005153.

4. Flisser A, Sarti E, Lightowlers M, Schantz P. Neurocysticercosis: regional status, epidemiology, impact and control measures in the Americas. Netherlands: Elsevier B.V; 2003. p. 43-51.

5. Rajshekhar V, Jeyaseelan L. Seizure outcome in patients with a solitary cerebral cysticercus granuloma. Neurology. 2004;62(12):2236-40.

6. Gulati S, Jain P, Sachan D, Chakrabarty B, Kumar A, Pandey RM, et al. Seizure and radiological outcomes in children with solitary cysticercous granulomas with and without albendazole therapy: A retrospective case record analysis. Epilepsy Research. 2014;108(7):1212-20.

7. Singh AK, Garg RK, Gupta RK, Malhotra HS, Agrawal GR, Husain N, et al. Dynamic contrast-enhanced (DCE) MRI derived kinetic perfusion indices may help predicting seizure control in single calcified neurocysticercosis. Magnetic Resonance Imaging. 2018;49:55-62.

8. Singh AK, Garg RK, Rizvi I, Malhotra HS, Kumar N, Gupta RK. Clinical and neuroimaging predictors of seizure recurrence in solitary calcified neurocysticercosis: A prospective observational study. Epilepsy Research. 2017;137:78-83.

9. Murthy JMK, Seshadri V. Prevalence, clinical characteristics, and seizure outcomes of epilepsy due to calcific clinical stage of neurocysticercosis: Study in a rural community in south India. Epilepsy & Behavior. 2019;98(Pt A):168-72.

10. Bustos JA. Calcified neurocysticercosis: risk factors for calcification and associated factors for seizure relapse: Johns Hopkins University; 2020.

11. Del Brutto OH, Campos X. Discontinuation of antiepileptic drugs in patients with calcified neurocysticercosis. Journal of epilepsy. 1996;9(4):231-3.

12. Singh G, Bawa J, Chinna D, Chaudhary A, Saggar K, Modi M, et al. Association between epilepsy and cysticercosis and toxocariasis: A population‐based case–control study in a slum in India. Epilepsia. 2012;53(12):2203-8.

13. Prasad A, Gupta RK, Pradhan S, Tripathi M, Pandey CM, Prasad KN. What triggers seizures in neurocysticercosis? A MRI-based study in pig farming community from a district of North India. Parasitology International. 2007;57(2):166-71.

14. Prasad KN, Prasad A, Gupta RK, Nath K, Pradhan S, Tripathi M, et al. Neurocysticercosis in patients with active epilepsy from the pig farming community of Lucknow district, north India. Transactions of the Royal Society of Tropical Medicine and Hygiene. 2009;103(2):144-50.

15. Nicoletti A, Bartoloni A, Sofia V, Bartalesi F, Chavez JR, Osinaga R, et al. Epilepsy and Neurocysticercosis in Rural Bolivia: A Population‐based Survey. Epilepsia. 2005;46(7):1127-32.

16. Cruz ME, Schantz PM, Cruz I, Espinosa P, Preux PM, Cruz A, et al. Epilepsy and neurocysticercosis in an Andean community. International journal of epidemiology. 1999;28(4):799-803.

17. Rajshekhar V, Raghava MV, Prabhakaran V, Oommen A, Muliyil J. Active epilepsy as an index of burden of neurocysticercosis in Vellore district, India. Neurology. 2006;67(12):2135-9.

18. Garvey BT, Moyano LM, Ayvar V, Rodriguez S, Gilman RH, Gonzalez AE, et al. Neurocysticercosis among People Living Near Pigs Heavily Infected with Cysticercosis in Rural Endemic Peru. The American journal of tropical medicine and hygiene. 2018;98(2):558-64.

19. Carpio A, Escobar A, Hauser WA. Cysticercosis and Epilepsy: A Critical Review. Epilepsia. 1998;39(10):1025-40.

20. Monteiro L, Almeida-Pinto J, Stocker A, Sampaio-Silva M. Active neurocysticercosis, parenchymal and extraparenchymal: a study of 38 patients. Journal of neurology. 1993;241(1):15.

21. Marcin Sierra M, Arroyo M, Cadena Torres M, Ramírez Cruz N, García Hernández F, Taboada D, et al. Extraparenchymal neurocysticercosis: Demographic, clinicoradiological, and inflammatory features. PLoS neglected tropical diseases. 2017;11(6):e0005646.

22. Serpa JA, Graviss EA, Kass JS, White JAC. Neurocysticercosis in Houston, Texas: an update. Medicine. 2011;90(1):81-6.

23. Shandera WX, White JAC, Chen JC, Diaz P, Armstrong R. Neurocysticercosis in Houston, Texas. A report of 112 cases. Medicine. 1994;73(1):37-52.

24. Monteiro L, Coelho T, Stocker A. Neurocysticercosis--a review of 231 cases. Germany1992. p. 61-5.

25. Sáenz B, Ruíz-Garcia M, Jiménez E, Hernández-Aguilar J, Suastegui R, Larralde C, et al. Neurocysticercosis: Clinical, Radiologic, and Inflammatory Differences Between Children and Adults. The Pediatric Infectious Disease Journal. 2006;25(9):801-3.

26. Auditeau E, Moyano LM, Bourdy G, Nizard M, Jost J, Ratsimbazafy V, et al. Herbal medicine uses to treat people with epilepsy: A survey in rural communities of northern Peru. Journal of Ethnopharmacology. 2018;215:184-90.

27. Colli BO, Carlotti JCG, Assirati JJA, Machado HR, Valença M, Amato MCM. Surgical treatment of cerebral cysticercosis: long-term results and prognostic factors. Neurosurgical focus. 2002;12(6):e3.

28. Figueroa JJ, Davis LE, Magalhaes A. Extraparenchymal Neurocysticercosis in Albuquerque, New Mexico. Journal of Neuroimaging. 2011;21(1):38-43.

29. Sharma BS, Sawarkar DP, Verma SK. Endoscopic Management of Fourth Ventricle Neurocysticercosis: Description of the New Technique in a Case Series of 5 Cases and Review of the Literature. World Neurosurgery. 2019;122:e647-e54.

30. Bandres JC, White AC, Samo T, Murphy EC, Harris RL. Extraparenchymal Neurocysticercosis: Report of Five Cases and Review of Management. Clinical Infectious Diseases. 1992;15(5):799-811.

31. Lobato RD, Lamas E, Portillo JM, Roger R, Esparza J, Rivas JJ, et al. Hydrocephalus in cerebral cysticercosis. Pathogenic and therapeutic considerations. Journal of neurosurgery. 1981;55(5):786.

32. Nash TE, O'Connell EM, Hammoud DA, Wetzler L, Ware JM, Mahanty S. Natural History of Treated Subarachnoid Neurocysticercosis. The American journal of tropical medicine and hygiene. 2020;102(1):78-89.

33. Colli BO, Martelli N, Assirati JJA, Machado HR, de Vergueiro Forjaz S. Results of surgical treatment of neurocysticercosis in 69 cases. Journal of neurosurgery. 1986;65(3):309-15.

34. Sotelo J, Marin C. Hydrocephalus secondary to cysticercotic arachnoiditis. A long-term follow-up review of 92 cases. Journal of neurosurgery. 1987;66(5):686.

35. Kelley R, Duong DH, Locke GE. Characteristics of ventricular shunt malfunctions among patients with neurocysticercosis. Neurosurgery. 2002;50(4):757-62.

36. Goel RK, Ahmad FU, Vellimana AK, Suri A, Chandra PS, Kumar R, et al. Endoscopic management of intraventricular neurocysticercosis. Journal of clinical neuroscience. 2007;15(10):1096-101.

37. Husain M, Jha DK, Rastogi M, Husain N, Gupta RK. Neuro-endoscopic management of intraventricular neurocysticercosis (NCC). Acta neurochirurgica. 2007;149(4):341-6.

38. Proaño JV, Torres-Corzo J, Rodríguez-Della Vecchia R, Guizar-Sahagun G, Rangel-Castilla L. Intraventricular and subarachnoid basal cisterns neurocysticercosis: a comparative study between traditional treatment versus neuroendoscopic surgery. Child's nervous system. 2009;25(11):1467-75.

39. Rangel-Castilla L, Serpa JA, Gopinath SP, Graviss EA, Diaz-Marchan P, White AC, Jr. Contemporary Neurosurgical Approaches to Neurocysticercosis. American Journal of Tropical Medicine and Hygiene. 2009;80(3):373-8.
