# Supplementary material for: CystiHuman: A model of human neurocysticercosis
Source: PLoS Comput Biol. 2022 May 19;18(5):e1010118. doi: 10.1371/journal.pcbi.1010118 (PMC9159625; doi:10.1371/journal.pcbi.1010118)
Supplement: S3 Text — Table A in S3 Text: 2016 estimate of the case fatality ratios for active epilepsy in selected American countries. Table B in S3 Text Parameters describing short-term movements to and from the villages. Table C in S3 Text: Percentage of Peru and Piura population, by age range, that died or emigrated out of the country between 2011 and 2016. Table D in S3 Text: Percentage of Piura population, by age range, that changed district within Peru between 2011 and 2016. Table E in S3 Text: Estimated yearly rate of emigration out of Gates 2 villages (movement out of the village of origin but within the country). Table F in S3 Text: 2016 Piura population, by age range, that lived in another district or abroad in 2011, as a share of 2011 Piura population in age range. Table G in S3 Text: Estimated yearly rate of immigration into Gates 2 villages. Table H in S3 Text: Immigration into Piura: actual for 2011–2016 and revised values. Table I in S3 Text: Emigration from a Piura district: actual for 2011–2016 and revised values. Table J in S3 Text: Deaths (plus emigration to another country): actual for 2011–2016 and revised values. Table K in S3 Text: Newcomers–immigrants and births (number of individuals and share of all newcomers). Fig A in S3 Text: Percentage of deaths among surgical cases. Fig B in S3 Text: Percentage of deaths among severe cases. Fig C in S3 Text: Representation of the ‘reality’ of demographic changes. Fig D in S3 Text: Simplification of people’s history when they have lived in different places with various levels of risks before coming to the village. Fig E in S3 Text: Simplified representation of demographic changes. (DOCX) [file pcbi.1010118.s003.docx]

Supporting information 3 – Demographic data (including NCC-related deaths) and methodology

This section details data and methods related to demographic changes (NCC-related and natural), including:

1. Data on death rates for individuals with NCC (deaths from active epilepsy, surgical treatment of ICH/hydrocephalus, and untreated ICH/hydrocephalus).
2. ‘Natural’ death rates, by age range.
3. Birth rates.
4. Emigration rates, by age range.
5. Immigration rates, by age range and origin.
6. Detailed description of the methodology used to model demographic changes in the population using data on births, deaths and human movements.

# NCC-related deaths

## Deaths from active epilepsy

A majority of individuals with symptomatic NCC present with epilepsy. In Peru, according to a study prepared for the Global Burden of Disease 2016 [1], the yearly mortality from active epilepsy was around 0.13% (185/140,175). Estimates for different countries are provided in Table A in S3 Text.

**Table A: 2016 estimate of the case fatality ratios for active epilepsy in selected American countries**

| **Country** | **Case fatality ratio for active epilepsy** |
| --- | --- |
| Brazil | 0.30% [0.23-0.43%] |
| Peru | 0.13% [0.07-0.58%] |
| USA | 0.18% [0.14-0.22%] |
| Chile | 0.36% [0.19-1.58%] |
| Mexico | 0.27% [0.22-0.36%] |

*Sources: [1] for the raw data, [2] for the computation of the confidence interval*

## Deaths from intracranial hypertension (ICH) or hydrocephalus

Synthesizing data about deaths from ICH or hydrocephalus is hampered by large heterogeneity between studies, in their focus: surgical cases, ICH/hydrocephalus cases, cases with giant subarachnoid cysts, etc.; their follow-up: from operative mortality to over 5 years of follow-up; the country/region in which the deaths happened; and their date. Two values are of particular interest for the paper: 1) future death rates in Peru, for integration into model projections – these are unknown but may be approximated using current death rates in Latin America and 2) death rates in Latin America a few decades ago. These can be used, in particular, to estimate past deaths from NCC-related convulsions vs. ICH/hydrocephalus as a function of *ξ* and compare these estimates with death records (see 1.3).

We used two approaches: focusing on deaths in Latin America among surgical cases only, with the limitation that few studies fit the criteria, despite inclusion of a variety of time periods; and relaxing the condition on the type of case studied, with the hope of being able to undertake a meta-regression on the time period during which patient treatment and follow-up took place. The two approaches and their limitations are discussed below.

Fig A in S3 Text focuses on deaths among surgical cases only, and in Latin America. In this context, average mortality after NCC-related surgery was 34% [22-45%] on average for the period considered (1945 to 2001). Studies are ordered by the middle of the period of data collection (e.g., for Lopes (1971), the data collection period is 1945-1968 and the middle of that period is 1956.5). Follow-up (FU) is deemed “short” when it is, on average, inferior to 2 years, “long” when it is 5 years or more, and “median” otherwise. Unfortunately, with only 5 studies, one with of which has a very low sample size, and very different dates but all 20 or more years ago, this estimated death rate is only indicative and not very useful for future projections.

**Fig A: Percentage of deaths among surgical cases**

**
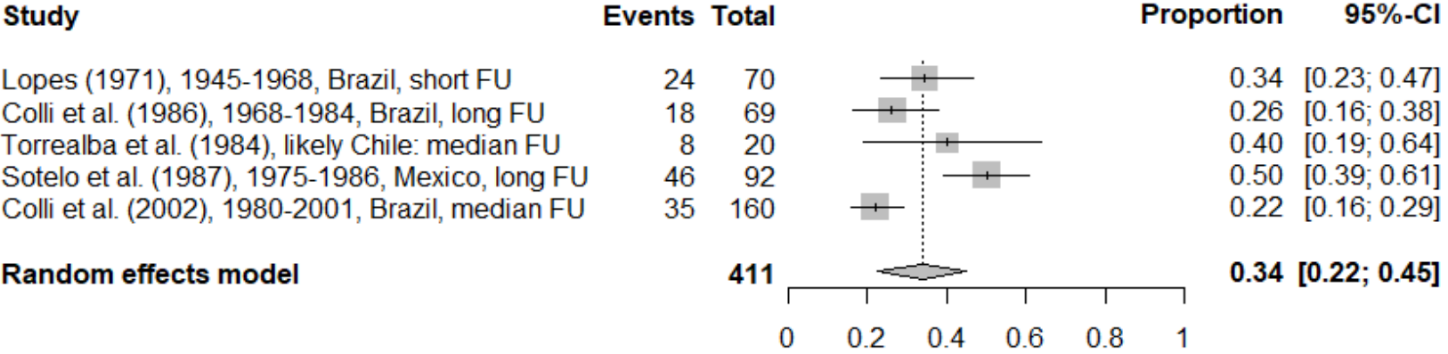
**

*Source:[3-7]*

The second approach was to include more studies, beyond surgical cases (hypertensive cases, cases with giant subarachnoid cysts, or other severe presentations). If studies with very small sample sizes (15 or less) are excluded, we can include a total of 7 studies. In Fig B in S3 Text, these are classified by country then, within a given country, by the median year of data collection.

**Fig B: Percentage of deaths among severe cases**


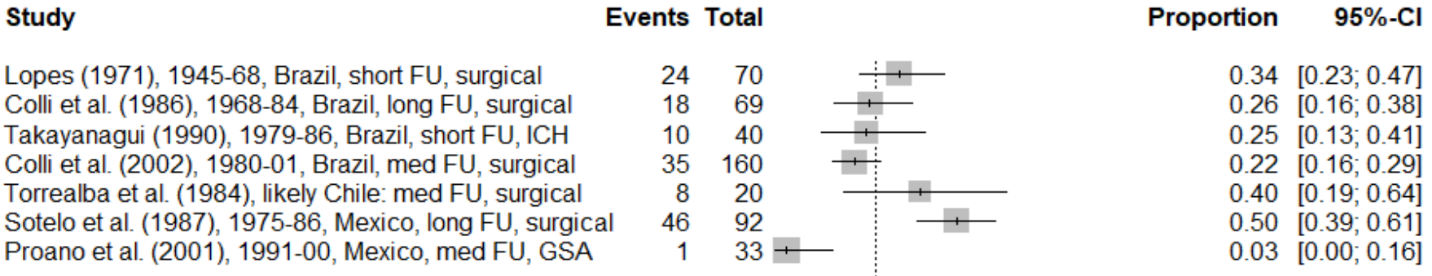
*Sources: [3-9], GSA refers to cases with giant subarachnoid cysts. In [8], ICH cases correspond to cases classified as having intracranial hypertension as a primary presentation.*

We can see that, within Mexico and within Brazil, death rates appear to decrease over time. We undertook a meta-regression analysis on studies from Brazil. The date of the cases had a significant impact on the likelihood of death, with a 0.36% [0.25-0.47%] decline every year in the probability of dying. Extrapolating the risk of death to 2020, the expected case fatality ratio would be 11%, while in 1950, it would be 36%. It is uncertain how much this analysis could be extrapolated to Peru. However, this result is at least compatible with discussions with clinical experts from Peru (H.G. & J.B.) that suggest that death rates today are likely around 10% or less.

We will therefore use 10% for future projections of death rates in clinical care settings in Peru. Meanwhile, the results of the meta-analysis and meta-regression will be used to model/analyze past data on ICH/hydrocephalus deaths among NCC cases.

Some individuals with ICH/hydrocephalus never go to hospital. They may have, in some cases, less serious disease than those that do (e.g., intermittent blockage of the cerebrospinal fluid rather than a constant increase in intracranial pressure). However, not going to hospital is still likely to prove deadly. In the absence of better data, we consider that surgically treated cases, even at a time when techniques were less effective than today, were less likely to die than individuals that never presented to hospital. We therefore use a bracket of [36%-100%] for the probability for an untreated NCC cases with ICH/hydrocephalus to die.

Regarding the timing of death, [3-5, 7] provide information regarding 1- and 2-year survival rates, and information on survival at 3 years (and more) is available in [3-5]. Based on this information, almost all deaths associated with surgery take place within the first 5 years, with approximately 64% taking place during the first year, 77% during the first two years, and 95% during the first three years (this is based on a weighted average using survival rates at 1, 2, 3 and 5 years for the studies with the corresponding information. An unweighted average would have found virtually the same figures. However, there is important heterogeneity between studies, and these figures are only indicative of the expected spread in the timing of deaths).

# Short-term movements

Short-term movements to and from the villages are represented through a rate of travel from other villages into the village, and the designation of individuals that are “frequent travelers” (at most one per household) and the definition of the frequency and duration of their travels. These figures were taken from earlier iterations of CystiAgent [10] and are the result of field surveys.

**Table B: Parameters describing short-term movements to and from the villages**

| **Parameter** | **Value** | **Meaning** |
| --- | --- | --- |
| *travelProp* | 0.423 | % of households with a frequent traveler [10] |
| *travelFreq* | 0.125 (every 8 weeks) | Frequency of trips by frequent travelers [10] |
| *travelDuration* | 1.75 weeks | Average duration of trips by frequent travelers [10] |
| *travelIncidence* | 2.3 10^-4^ | Incidence of human taeniasis at destination [11] |

*Sources: [10, 11]*

# ‘Natural’ death rates, by age range

The following sections on death, birth, emigration and immigration rates are common with the revised version of CystiAgent [10]. They are reproduced here for ease of reference.

Country level and survey-specific data can provide insights on ‘natural’ population changes. Overall deaths, by age range, can be deduced from country-level data (2019 World Population Prospects [12]) and Piura specific data (2017 INEI [13], using information on the region an individual lived in 5 years ago, combined with [14] for regional population by age range in 2016 and 2011).

**Table C: Percentage of Peru and Piura population, by age range, that died or emigrated out of the country between 2011 and 2016**

| Age range | 0-4 | 5-9 | 10-14 | 15-19 | 20-24 | 25-29 | 30-34 | 35-39 |
| --- | --- | --- | --- | --- | --- | --- | --- | --- |
| All Peru | 3.1% | 3.4% | 4.6% | 4.3% | 4.1% | 1.8% | 0.4% | 0.5% |
| Piura region | 1.9% | 1.9% | 3.4% | 3.8% | 4.2% | 4.1% | 4.2% | 4.1% |
| Age range | 40-44 | 45-49 | 50-54 | 55-59 | 60-64 | 65-69 | 70-74 | 75+ |
| All Peru | 0.8% | 1.7% | 1.9% | 1.3% | 2.3% | 6.0% | 11.2% | 47.4% |
| Piura region | 4.5% | 4.9% | 5.3% | 6.6% | 9.1% | 13.4% | 19.6% | 42.3% |

*Sources: [12-14]*

Within-country movement is accounted for and not confused with deaths. Emigration out of the country is harder to track, which is why Table C in S3 Text does not distinguish it from deaths. Our knowledge of population movements at a more granular level (e.g. province, district, or village-level) is more limited and we were therefore not able to estimate death rates at these levels.

# Birth rates

For the 2011 to 2016 period, the average birth rate was: 18.0 per thousand inhabitants per year for Peru as a whole (2019 World Population Prospects [12]), and 20.3 and 23.1 per thousand for the Piura region and the Ayabaca province within the Piura region respectively (INEI [13]). Figures for rural Piura for 2012 using the Demographic and Health Survey [15] are similar to those for the Piura region overall (20.9 per thousand inhabitants per year). Data from prior surveys in target villages show similar birth rates, but the precision is lower given the size of the subsample aged one year and younger.

# Emigration rates, by age range

Individuals that emigrate affect the age structure of the village population. Further, contrary to people that die, individuals that emigrate may still benefit, after emigration, from the long-term effects of interventions that took place while they were living in the village. Emigration out of the country cannot easily be distinguished from death, but within-country movement can.

There are two sources of information for within-country movement. National surveys [13] provide extensive information as to how many individuals living in a region in a given year were living in the same district 5 years ago. This does not include individuals that changed village within a district. Meanwhile, endemic villages targeted for past studies have sometimes been visited twice, as was the case for the study described in (4) (we will refer to this study as “GATES2” in what follows). This enables us to estimate the rate of movement out of specific endemic villages, excluding emigration to other countries or deaths, using the estimates in Table D in S3 Text. Given the low samples for higher ages and the fact that we are combining data from two different data sources, data for 60 years and above are not reliable.

**Table D: Percentage of Piura population, by age range, that changed district within Peru between 2011 and 2016**

| Age range | 0-4 | 5-9 | 10-14 | 15-19 | 20-24 | 25-29 | 30-34 | 35-39 |
| --- | --- | --- | --- | --- | --- | --- | --- | --- |
| Piura region | 7.3% | 7.1% | 12.8% | 18.1% | 15.3% | 12.6% | 10.7% | 9.2% |
| Age range | 40-44 | 45-49 | 50-54 | 55-59 | 60-64 | 65-69 | 70-74 | 75+ |
| Piura region | 11.2% | 7.6% | 7.4% | 6.8% | 6.5% | 5.7% | 5.2% | 4.2% |

*Sources: [12-14]*

**Table E: Estimated yearly rate of emigration out of GATES2 villages (movement out of the village of origin but within the country)**

| Age range | 0-4 | 5-9 | 10-14 | 15-19 | 20-24 | 25-29 |
| --- | --- | --- | --- | --- | --- | --- |
| % from village moved within country | 4.2% | 3.1% | 0.5% | 3.9% | 4.7% | 4.3% |
| Age range | 30-34 | 35-39 | 40-44 | 45-49 | 50-54 | 55-59 |
| % from village moved within country | 3.8% | 0.8% | 1.5% | 0.8% | 0.3% | 0.2% |

*Sources: [ 4 ] and Table C in S3 Text*

*Note: Not all data were collected at the exact same time, but most were collected between early 2007 (for the baseline census) and late 2007 (for the second visit).*

Data for Piura (computed over 5 years) are smoother and more reliable than data from the GATES2 villages (computed over 1 year). Both tables are not fully comparable as Table D in S3 Text does not include mobility within a district. However, overall, figures are similar enough (an estimated 10.3% of individuals living in Piura in 2011 had moved by 2016 vs. 12-13% of the GATES2 village population if we use the yearly emigration rate found in Table E in S3 Text for 5 years in a row). Piura data was therefore used for the purpose of the model.

# Immigration rates, by age range and origin

Again, immigration rates are reasonably well-known for the Piura region. In total, the 2016 Piura population that, in 2011, lived in another district than their 2016 district, represented 12.6% of the 2011 Piura population.

**Table F: 2016 Piura population, by age range, that lived in another district or abroad in 2011, as a share of 2011 Piura population in age range**

| Age range | 0-4 | 5-9 | 10-14 | 15-19 | 20-24 | 25-29 | 30-34 | 35-39 |
| --- | --- | --- | --- | --- | --- | --- | --- | --- |
| Piura region | 7.2% | 6.8% | 9.5% | 13.1% | 13.9% | 12.7% | 11.1% | 7.2% |
| Age range | 40-44 | 45-49 | 50-54 | 55-59 | 60-64 | 65-69 | 70-74 | 75+ |
| Piura region | 8.5% | 7.5% | 7.0% | 6.2% | 5.7% | 4.9% | 4.4% | 3.6% |

*Sources: [12-14]*

Meanwhile, we also have an estimate for GATES2 villages *(4)*. The immigration rate is 3.3% per year and 17.6% over 5 years, including movement between two villages within the same study. Though this is higher than for the Piura region, figures are similar enough and Piura figures should be more reliable, hence this is what was used in the model.

**Table G: Estimated yearly rate of immigration into GATES2 villages**

| Age range | 0-4 | 5-9 | 10-14 | 15-19 | 20-24 | 25-29 | 30-34 | 35-39 |
| --- | --- | --- | --- | --- | --- | --- | --- | --- |
| % in age range that moved into village | 5.2% | 3.9% | 2.4% | 2.6% | 3.7% | 4.7% | 5.5% | 2.5% |
| Age range | 40-44 | 45-49 | 50-54 | 55-59 | 60-64 | 65-69 | 70-74 | 75+ |
| % in age range that moved into village | 2.9% | 2.8% | 2.0% | 2.8% | 0.7% | 1.7% | 1.2% | 1.5% |

*Sources: (4)*

The origin of these migrants is critical: they may bring new *T. solium* tapeworms into the village and, regarding neurocysticercosis, their infection rate, if very different from that of the village, could significantly affect average village rates. Current estimates are that 61% of immigrants came from urban localities. However, these are based on questionnaires in the control and intervention village field trials (5,6) the GATES2 trial regarding the village of origin of migrants, but the coding of answers appears very unreliable. Field studies are planned to obtain reliable figures.

# Methodology used to represent demographic changes

The methodology used to represent deaths associated with NCC is described in the main paper. These deaths add up to natural population changes: natural deaths, births, and population movements. This section describes how these natural changes are modelled.

The reality of natural demographic changes (Fig C in S3 Text) is complex: it includes births and deaths, emigration to different places (low, medium or high risk), and immigration into the village of individuals that often have a complex history (e.g., stays in multiple places with diverse risks, including possibly the village they are now moving into). Short-term travel to various destinations (already included in CystiAgent and not represented on the graph) adds to long-term trends.

**
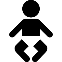

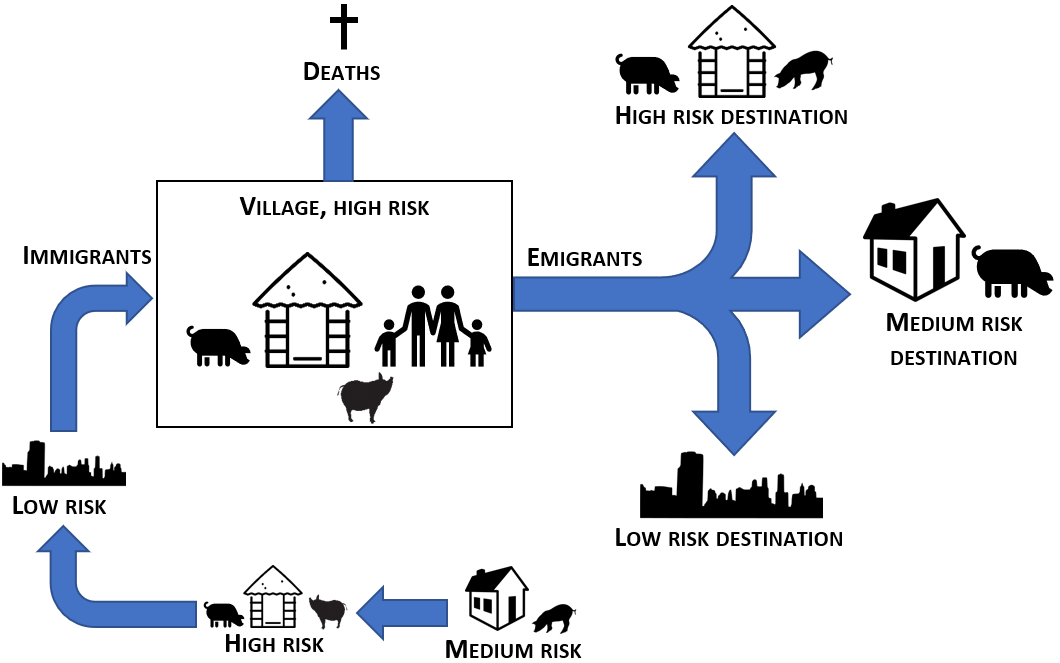
****Fig C: Representation of the ‘reality’ of demographic changes**

**Births**

It is proposed to model natural demographic changes as follows:

Deaths are modelled through a probability of dying applied to each age range (to which NCC-related risk of death is added if needed). Individuals that die are then removed from the simulation.

Meanwhile, for the purpose of modelling what takes place within the target villages, emigration is equivalent to death, and removing emigrants from the simulation could be enough. However, the benefits (fewer cases of epilepsy, ICH/hydrocephalus or deaths) of interventions to control/eliminate taeniasis and cysticercosis are only felt after a few years (over a decade for ICH or hydrocephalus). Hence, individuals that have benefited from an intervention may have already moved out, and neglecting the benefits accrued by emigrants could lead to significant underestimates of the impact of an intervention.

Emigrants may go to different places (large cities, small villages) and face diverse levels of risk there. What matters, however, is not the total risk faced by emigrants but the risk brought about in their life by their time in the village (and how this may be reduced by interventions). We therefore keep emigrants in the simulation but simplify the model by putting all emigrants in a fictitious zero-risk context (no eating of infected meat, or exposure or contribution to environmental contamination). The risk they face there only reflects the added risk brought about by their time in the village. This simplification assumes that, for the purpose of modelling emigrants, we can consider NCC-related risks as additive.

Immigrants affect the demographic composition and disease status of the village. For example, should all immigrants come from low-risk areas, they could significantly decrease the overall prevalence rate (if e.g., 61 per cent of them came from low-risk areas, and the rest came from high-risk areas, then we may find a disease prevalence of 24% if we do not account for immigration, but only 20% after accounting from it). The reality of immigration is complex, but we simplify it as follows:

1. Places of origin are modelled solely as “low-risk” (modelled by assuming individuals do not accumulate any cyst or contract taeniasis when living in such a locality) or “high-risk” (modelled by assuming individuals living in such a locality face the same risks as in the target villages).
2. Immigration from multiple places with different risk levels is simplified as follows: if N immigrants in a given age-range have spent x% of their past life in high-risk areas and (1-x%) in low-risk areas, they are equated with N × x% immigrants coming from high-risk areas exclusively, and N × (1-x%) coming from low-risk areas (see Fig D in S3 Text). This is acceptable if we can assume that 1) NCC-related risks are mostly additive, 2) high risk locations and low risk locations, for a given age range of immigrants, are randomly distributed (the timing of presence in high-risk locations affects the timing of infection hence the timing of symptoms).

**Fig D. Simplification of people’s history when they have lived in different places with various levels of risks before coming to the village**

| 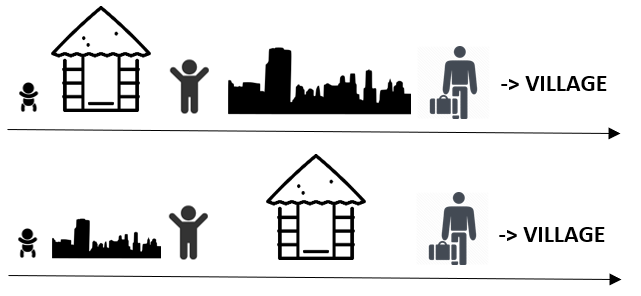 | becomes | 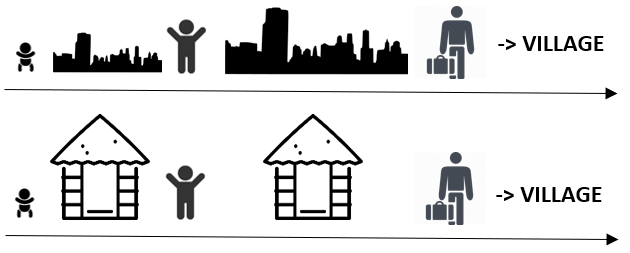 |
| --- | --- | --- |

Finally, births bring new inhabitants to the village with no infection status and age 0. The simplified demographic model therefore looks as follows:

**Fig E: Simplified representation of demographic changes**


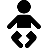

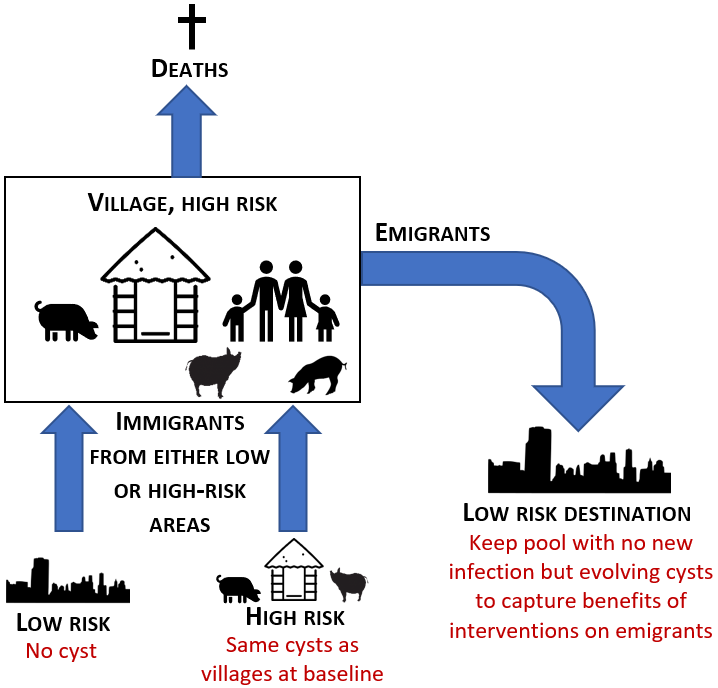


**Births**

**Population growth**

With the actual value of births, deaths, and migrations, it is expected that the population of the Piura region will grow by around 0.82% per year (based on INEI data for 2011 and 2016 [15]). Over 85 years of simulation (a duration similar to what is needed for individuals of all ages to accumulate NCC-related lesions), the population of the region would double. This is a problem for modelling, as if the model’s population is allowed to grow, either household size will reach unrealistic levels or new households will have to be created and located within growing village boundaries, in line with local practices (e.g., subdivision of the parents’ holding), which could prove complex. Further, using current birth and death rates over a 85 years’ period may significantly change the age structure of the population.

Instead, we propose to tweak the current values of birth, death, emigration and immigration rates so that: i) population by age range remain constant, ii) overall numbers of emigrants, births, deaths and immigrants (total and by age range) be as close as possible to the actual value.

Let us note b the birth rate (births / total population), e the emigration rate, i the immigration rate, d the death rate, p_0-4_ the proportion of the total population aged 0 to 4 and e_0-4_ (and similar notations) the share of the 0 to 4 years old population that emigrates over a 5-year period. Tweaked values will be noted b’, e’, i’ etc. We keep the overall population constant and minimize (d’_0-4_ – d_0-4_)^2^ + (e’_0-4_ – e_0-4_)^2^ + (i’_0-4_ – i_0-4_)^2^ and similar values for other age ranges. This leads to the following equations:

To keep the population constant:

- b’ = p_0-4_
- 1 – p_5-9_ / p_0-4_ = d’_0-4_ + e’_0-4_ – i’_0-4_, …. 1 – p_80+_ / (p_75-79_ + p_80+_) = d’_75+_ + e’_75+_ – i’_75+_

To minimize the difference between the new rates and the actual rates:

- 3 (d’_0-4_ + e’_0-4_) = d_0-4_ + e_0-4_ + 2 (1 – p_5-9_ / p_0-4_) + 2 i_0-4_
- …
- 3 (d’_75+_ + e’_75+_) = d_75+_ + e_75+_ + 2 (1 – p_80+_ / (p_75-79_ + p_80+_)) + 2 i_75+_

And:

- 2 d’_0-4_ + e’_0-4_ = d_0-4_ + (1 – p_5-9_ / p_0-4_) + i_0-4_
- …
- 2 d’_75+_ + e’_75+_ = d_75+_ + (1 – p_80+_ / (p_75-79_ + p_80+_)) + i_75+_

The ‘actual’ (best estimates for the Piura region) and revised (values closest to reality while also maintaining the population constant) values of birth, “immigration” (movement into the village), “emigration” (movement out of the village) and death rates are given below.

Birth rates: 0-4 years old in 2016 represented 10.24% of the total 2011 Piura population. However, the 0-4 years old population in 2011 was 10.66% of the total population so, to ensure stability, 0-4 years old children 5 years later should represent 10.66% of the Piura population. To maintain the population structure, there should be a slightly higher birth rate than there actually is.

Immigration rates: to maintain the current population structure, there should be slightly more immigrants at the lowest ages (below 15 years old) but fewer immigrants at older ages than in reality. Overall, newcomers to Piura districts represented 9.3% of the Piura population, whereas the adjusted figure is 7.8%.

**Table H: Immigration into Piura: actual for 2011-2016 and revised values**

| Age range | 0-4 | 5-9 | 10-14 | 15-19 | 20-24 | 25-29 | 30-34 | 35-39 | 40-44 | 45-49 | 50-54 | 55-59 | 60-64 | 65-69 | 70-74 | 75+ |
| --- | --- | --- | --- | --- | --- | --- | --- | --- | --- | --- | --- | --- | --- | --- | --- | --- |
| Actual | 7.2% | 6.8% | 9.5% | 13.1% | 13.9% | 12.7% | 11.1% | 9.5% | 8.5% | 7.5% | 7.0% | 6.2% | 5.7% | 4.9% | 4.4% | 3.6% |
| Adjusted | 7.8% | 7.6% | 10.8% | 12.3% | 11.1% | 11.5% | 9.7% | 6.1% | 5.1% | 4.5% | 3.0% | 0.1% | 0.6% | 2.0% | 1.2% | 0.0% |

*Sources: [12-14] and authors’ computations*

Emigration rates: to maintain the current population structure, there should be slightly fewer emigrants at the lowest ages (below 15 years old) but more numerous emigrants at older ages than in reality. Overall, an estimated 10.3% of the Piura population left between 2011 and 2016, and the adjusted emigration rate is 12.0%.

**Table I: Emigration from a Piura district: actual for 2011-2016 and revised values**

| Age range | 0-4 | 5-9 | 10-14 | 15-19 | 20-24 | 25-29 | 30-34 | 35-39 | 40-44 | 45-49 | 50-54 | 55-59 | 60-64 | 65-69 | 70-74 | 75+ |
| --- | --- | --- | --- | --- | --- | --- | --- | --- | --- | --- | --- | --- | --- | --- | --- | --- |
| Actual | 7.3% | 7.1% | 12.8% | 18.1% | 15.3% | 12.6% | 10.7% | 9.2% | 11.2% | 7.6% | 7.4% | 6.8% | 6.5% | 5.7% | 5.2% | 4.2% |
| Adjusted | 6.6% | 6.3% | 11.5% | 18.9% | 18.1% | 13.8% | 12.2% | 12.6% | 11.8% | 10.6% | 11.5% | 13.0% | 11.6% | 8.6% | 8.4% | 7.8% |

*Sources: [12-14] and authors’ computations*

Death rates: to maintain the current population structure, there should be slightly fewer deaths at the lowest ages (below 15 years old) but more numerous deaths at older ages than in reality. Overall, an estimated 5.0% of the Piura population died between 2011 and 2016, and the adjusted death rate is 6.6%.

**Table J: Deaths (plus emigration to another country): actual for 2011-2016 and revised values**

| Age range | 0-4 | 5-9 | 10-14 | 15-19 | 20-24 | 25-29 | 30-34 | 35-39 | 40-44 | 45-49 | 50-54 | 55-59 | 60-64 | 65-69 | 70-74 | 75+ |
| --- | --- | --- | --- | --- | --- | --- | --- | --- | --- | --- | --- | --- | --- | --- | --- | --- |
| Actual | 1.9% | 1.9% | 3.4% | 3.8% | 4.2% | 4.1% | 4.2% | 4.1% | 4.5% | 4.9% | 5.3% | 6.6% | 9.1% | 13.4% | 19.6% | 42.3% |
| Adjusted | 1.3% | 1.1% | 2.1% | 4.6% | 7.0% | 5.3% | 5.6% | 7.6% | 7.9% | 7.8% | 9.4% | 12.7% | 14.2% | 16.3% | 22.8% | 45.9% |

*Sources: [12-14] and authors’ computations*

The difference between expectations and reality for total emigrants, immigrants, and deaths is 1.4 to 1.6 percentage points (in absolute value), whereas for births it is 0.4 percentage points. If we add births and immigrants to build “newcomers”, then we can write the estimated number of newcomers for Piura every 5 years (using the 2011 population as a basis):

**Table K: Newcomers – immigrants and births (number of individuals and share of all newcomers)**

| Age range | 0-4 | 5-9 | 10-14 | 15-19 | 20-24 | 25-29 | 30-34 | 35-39 | 40-44 | 45-49 | 50-54 | 55-59 | 60-64 | 65-69 | 70-74 | 75+ |
| --- | --- | --- | --- | --- | --- | --- | --- | --- | --- | --- | --- | --- | --- | --- | --- | --- |
| Nb | 190271 | 14780 | 14498 | 20495 | 22825 | 18266 | 16279 | 12654 | 7356 | 5275 | 3995 | 2244 | 66 | 300 | 710 | 320 |
| Share | 57.6% | 4.5% | 4.4% | 6.2% | 6.9% | 5.5% | 4.9% | 3.8% | 2.2% | 1.6% | 1.2% | 0.7% | 0.0% | 0.1% | 0.2% | 0.1% |

*Sources: [14] for Piura 2011 population and adjusted birth and immigration rates (cf. Table H in S3 Text)*

With these new figures, demographics can be modelled as follows. At each time point and for each human:

- The individual dies with a probability equal to the death rate for his age range (and one time step). If an individual dies, s/he is removed from the simulation and a new individual is created. If the individual who died was the cook, the new individual becomes the cook.
- The individual (if not dead) emigrates with probability equal to the emigration rate for his age range (and one time step). If the individual emigrates, s/he is moved out of the village and no longer consumes infected pork, is affected by or affects environmental contamination. Meanwhile, a new individual is created to replace the emigrant. If the individual who emigrated was the cook, the new individual becomes the cook.
- Creation of a new individual: the new individual is created as a newborn or immigrant in a given age range according to the share of all newcomers that are expected to be in that age range. If the new individual is an immigrant, s/he is either i) considered as coming from a “low risk” area with probability p_low_ and from a “high risk” area otherwise. The share of immigrants coming to endemic villages from low-risk areas is badly known. Studies are planned to collect this information and all other information relevant to the model from the same community. In the meanwhile, the figure of 5% was used, i.e., assuming that migration from larger cities to smaller villages is limited.
- Immigrants coming from low-risk areas are created with 0 neurocysticercosis lesion and no taeniasis infection. The disease status of immigrants coming from high-risk areas is defined by i) taking a random individual in that age-range in the target village if the burn-in period has not yet ended or, after the end of the burn-in period: ii) taking one or several “pictures” of the target villages around the end of the burn-in period and modelling the state of new immigrants from high-risk areas on a random individual in that or those “pictures”, choosing an individual in the appropriate age-range.

# References

1. Beghi E, Giussani G, Nichols E, Abd-Allah F, Abdela J, Abdelalim A, et al. Global, regional, and national burden of epilepsy, 1990–2016: a systematic analysis for the Global Burden of Disease Study 2016. The Lancet Neurology. 2019;18(4):357-75.

2. Donner A, Zou GY. Closed-form confidence intervals for functions of the normal mean and standard deviation. Statistical methods in medical research. 2010;21(4):347-59.

3. Lopes PG. Tratamento cirúrgico da cisticircose da fossa craniana posterior Surgical treatment of cysticercosis in posterior cranial fossa. Academia Brasileira de Neurologia (ABNEURO); 1971. p. 76-92.

4. Colli BO, Carlotti JCG, Assirati JJA, Machado HR, Valença M, Amato MCM. Surgical treatment of cerebral cysticercosis: long-term results and prognostic factors. Neurosurgical focus. 2002;12(6):e3.

5. Colli BO, Martelli N, Assirati JJA, Machado HR, de Vergueiro Forjaz S. Results of surgical treatment of neurocysticercosis in 69 cases. Journal of neurosurgery. 1986;65(3):309-15.

6. Torrealba G, Del Villar S, Tagle P, Arriagada P, Kase CS. Cysticercosis of the central nervous system: clinical and therapeutic considerations. Journal of Neurology, Neurosurgery & Psychiatry. 1984;47(8):784-90.

7. Sotelo J, Marin C. Hydrocephalus secondary to cysticercotic arachnoiditis. A long-term follow-up review of 92 cases. Journal of neurosurgery. 1987;66(5):686.

8. Takayanagui OM. Neurocysticercosis. I. Clinical and laboratory course of 151 cases. Brazil1990. p. 1.

9. Proaño JV, Madrazo I, Avelar F, López-Félix B, Díaz G, Grijalva I. Medical Treatment for Neurocysticercosis Characterized by Giant Subarachnoid Cysts. The New England Journal of Medicine. 2001;345(12):879-85.

10. Pizzitutti F, Bonnet G, Gonzales-Gustavson EA, Gabriël S, Pan WK, Pray IW, et al. Non-local validated parametrization of an agent-based model of local-scale Taenia solium transmission in North-West Peru. Submitted.

11. Beam M, Spencer A, Fernandez L, Atto R, Muro C, Vilchez P, et al. Barriers to Participation in a Community-Based Program to Control Transmission of Taenia solium in Peru. The American journal of tropical medicine and hygiene. 2018;98(6):1748-54.

12. United Nations, Department of Economic and Social Affairs, Population Division. World Population Prospects 2019, Online Edition. Rev. 1. 2019.

13. Censos nacionales 2017: XII de poblacion, VII de vivienda y III de communidades indigenas, sistema de consulta de base de datos [Internet]. 2017 [cited August 2020]. Available from: <http://censos2017.inei.gob.pe/redatam/>.

14. Poblacion estimada por edades simples y grupos de edad, degun provincia y distrito, departamento de Piura [Internet]. [cited August 2020]. Available from: <http://www.minsa.gob.pe/estadisticas/estadisticas/Poblacion/PoblacionMarcos.asp?20>.

15. Perú Encuesta Demográfica y de Salud Familiar - ENDES 2012. In: INEI/Perú INdEeI-, editor. Lima, Perú: ICF; 2013.
