# Supplementary material for: CystiHuman: A model of human neurocysticercosis
Source: PLoS Comput Biol. 2022 May 19;18(5):e1010118. doi: 10.1371/journal.pcbi.1010118 (PMC9159625; doi:10.1371/journal.pcbi.1010118)
Supplement: S4 Text — Fig A in S4 Text: Brain cyst time step. Fig B in S4 Text: Human time step (Module 1): development of new (immature) cysts. Fig C in S4 Text: Human time step (Modules 2 & 3). Fig D in S4 Text: parameter range post calibration (Module 1). Fig E in S4 Text: parameter range post calibration (Module 2). (DOCX) [file pcbi.1010118.s004.docx]

Supporting information 4 – Flow charts and calibration details

## Flow charts

This section provides the flow charts for cysts and human time steps. Every new time step, cyst and human ages increase by one week. In addition, *t_seizure_* (time since last epileptic seizure, a state variable for cysts) also increases by one week if its prior value is not NA (no seizure so far).

Fig A in S4 Text represents the processes associated with a brain cyst. Processes associated with Module 1 (cyst stages) are represented in black, those associated with Module 2 (epilepsy symptoms) are in yellow, and those associated with Module 3 (ICH/hydrocephalus symptoms) in green.

**Fig A: Brain cyst time step**

**
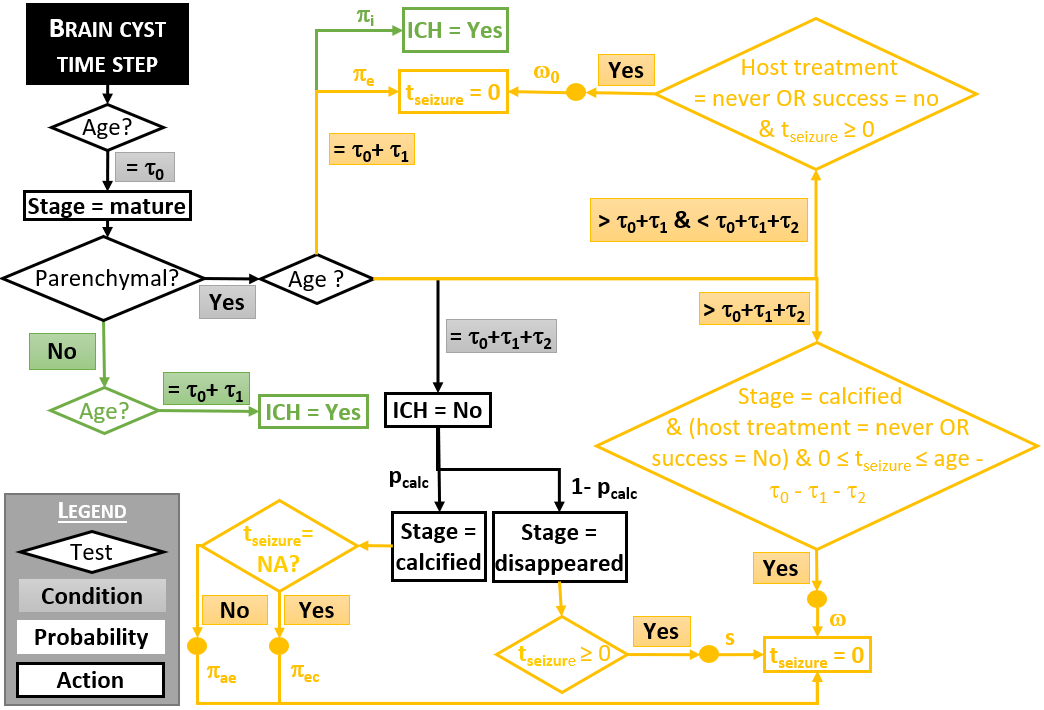
**

Fig B in S4 Text represents solely Module 1 of the human time step (development of new cysts).

**Fig B: Human time step (Module 1): development of new (immature) cysts**


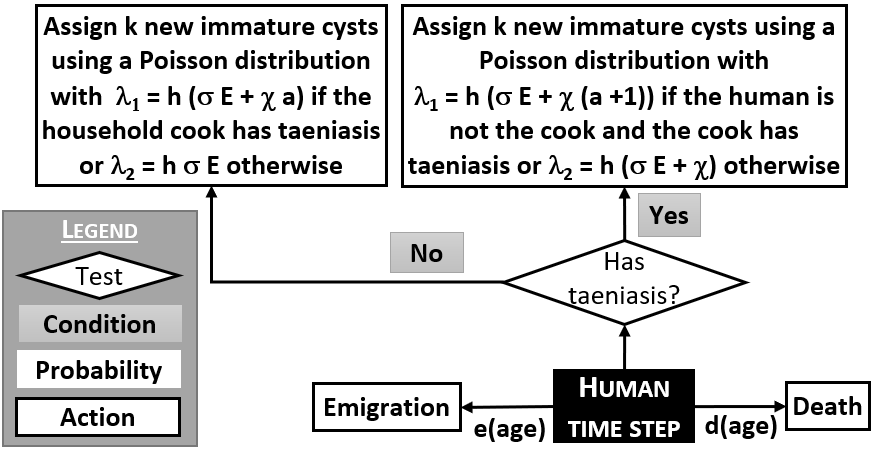


Fig C in S4 Text represents Modules 2 and 3 of the human time step. As with the cyst-related flow chart, Module 2 processes are represented in yellow, while Module 2 processes are in green.

**Fig C: Human time step (Modules 2 & 3)**

**
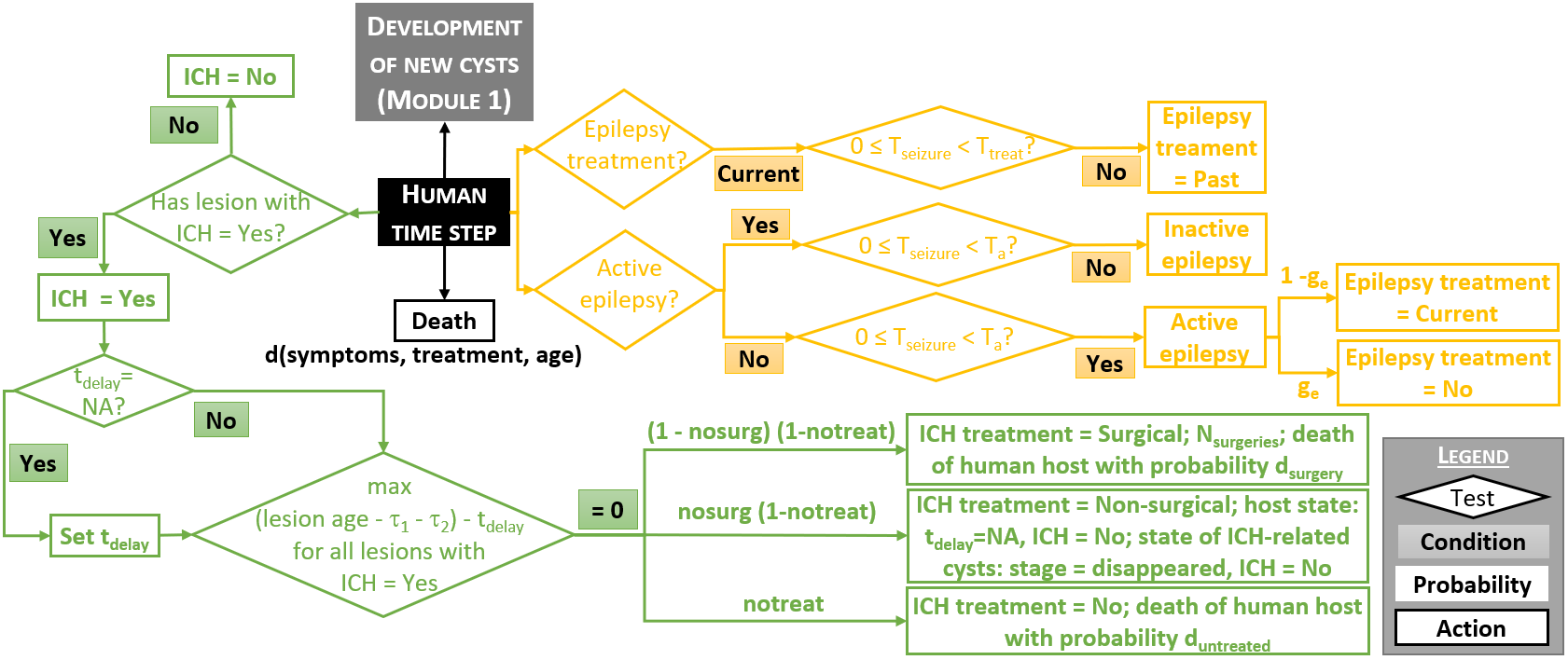
**

The box “development of new cysts (Module 1)” corresponds to the processes described in Fig B in S4 Text. In Modules 2 & 3, deaths linked to NCC symptoms (epilepsy and ICH) are added to ‘natural’ demographic processes included in Module 1.

## Calibration details

### Module 1 results

Fig D in S4 Text shows the range and median values for accepted parameters at the end of the calibration process for module 1. These results were obtained after three stages of calibration, beyond which new stages no longer improved the precision in model outputs and the calibration was stopped.

**Fig D: parameter range post calibration (Module 1)**

**
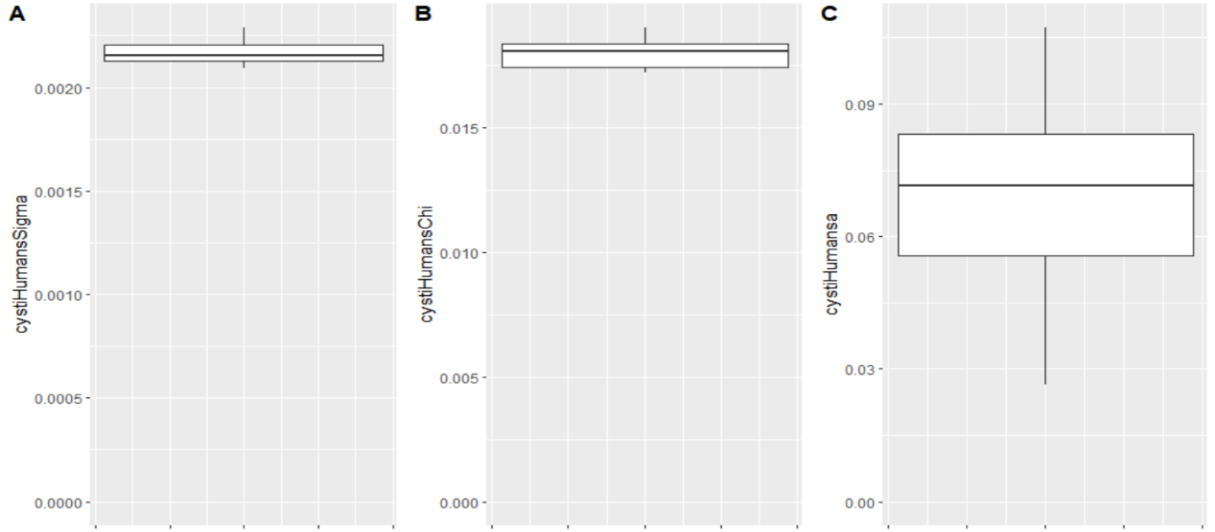
**

*a* = 0.0714

*χ* = 0.0180

*σ* = 0.00215

*The extreme values of the range of accepted parameters are quite different for a (the largest accepted value for this parameter is 3 times the minimum value) and very close for χ and σ (maximum over minimum = 1.1).*

Further, the target values are well approximated. Table A in S4 Text compares the target observables with the outcome of the simulation for each of the three villages using the median of accepted runs as the calibrated parameters. These simulated outcomes have been averaged over many runs to ensure confidence in the simulated average (e.g., a simulated average of 72.0% in the table means we are confident that the simulated average for this village is within the 71.95%-72.05% interval). The simulated average share of cases with one lesion is less than 5% different from the observables in all three villages, and the simulated share of cases with two lesions is less than 9% different from the observables. The distribution of cases by number of lesions is very stable across villages despite variations in village-level NCC prevalence rate, in line with the high stability seen across countries and communities (see S1 Text). The third observable was average NCC prevalence. It differs from village to village, and we sought to reproduce the average found across other similar communities through the average of the 3 villages, not values in individual villages. This approximation is very good (2.1% difference). In this simulation, between-village differences are driven mostly by differences in environmental contamination, themselves affected by taeniasis prevalence but also human density in the village. Calibration using actual values from multiple villages rather than proxies could help assess whether this influence of human density holds in practice, also helping validate the model.

**Table A: difference between model simulations and target values for the median of accepted runs**

| **Observable** | **Average % of cases with 1 lesion** | **Average % of cases with 2 lesions** | **Average NCC prevalence (18 years and older), as seen on CT scan** |
| --- | --- | --- | --- |
| Observed value | 72.5% | 16.9% | 20.0% |
| Simulated, village 515 | 72.0% | 18.4% | 19.7% |
| Simulated, village 566 | 74.8% | 17.0% | 13.9% |
| Simulated, village 567 | 75.5% | 17.1% | 22.1% |
| *Average 3 villages* | *74.6%* | *17.4%* | *19.6%* |

### Module 2 results

Fig E in S4 Text shows the result of the calibration. Four stages of calibration were undertaken before no further improvement in calibration outputs could be seen. At the end of the calibration, the parameter space had narrowed substantially for π*_e_* and π*_ec_*, less so for π*_ae_*.

**Fig E: parameter range post calibration (Module 2)**


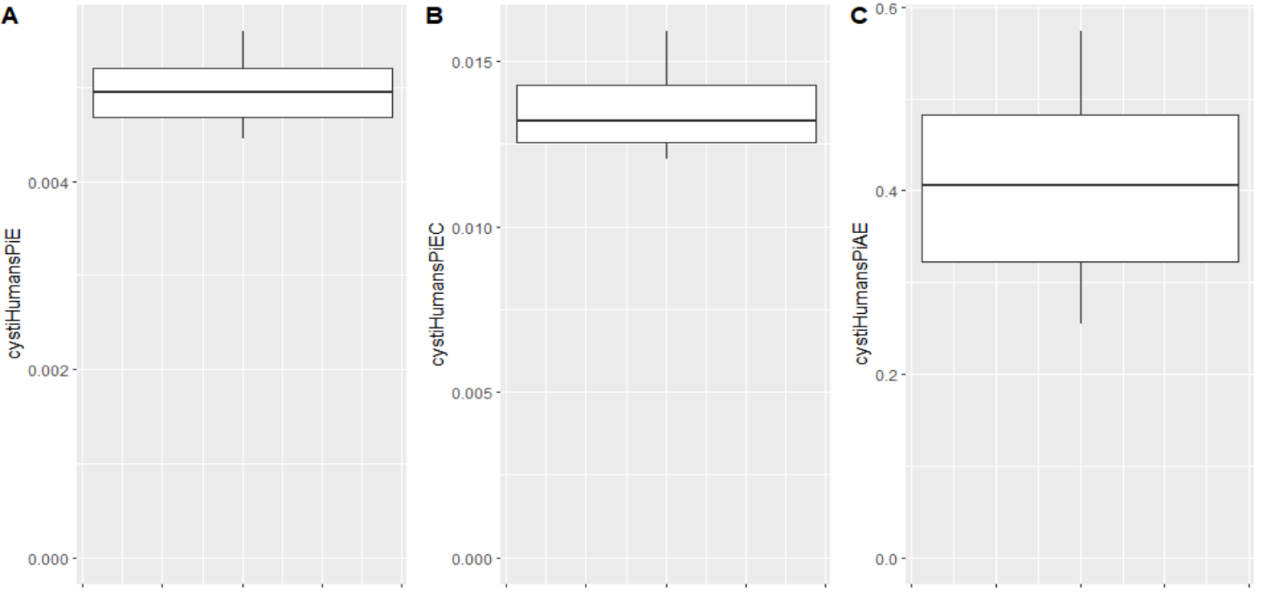


π*_ae_* = 0.406

*π_ec_* = 0.0132

π*_e_* = 0.0050

*The extreme values of the range of accepted parameters are very different for π_ae_ (the largest accepted value for this parameter is 2.3 times the minimum value) and reasonably close for π_e_ and π_ec_ (maximum over minimum: 1.3).*

The fit between simulated values using the selected parameters (median of accepted runs) and actual observables is good. The relative difference between simulated and observed values is lower than 8%, 13% and 4% in any village for the first, second and third observables respectively (Table B in S4 Text).

**Table B: difference between model simulations and target values for the median of accepted runs**

| **Observable** | **% of NCC cases with NCC-driven epilepsy** | **% of parenchymal active epilepsy NCC cases that are non-calcified** | **% of calcified parenchymal NCC cases with epilepsy that have (long-term) active epilepsy** |
| --- | --- | --- | --- |
| Observed value | 2.1% | 15.9% | 66.2% |
| Simulated, village 515 | 2.3% | 18.0% | 64.4% |
| Simulated, village 566 | 2.1% | 17.7% | 65.4% |
| Simulated, village 567 | 2.1% | 15.0% | 65.5% |
| *Average 3 villages* | *2.1%* | *16.3%* | *65.2%* |

### Module 3 results

This module was calibrated manually, and we found that *ξ* = 0.0108 and *π_i_* = 0.00083 were the parameters that best approximated the expected result. The outputs of the model for these parameter values are provided in Table C in S4 Text. The difference between simulations and observables is less than 5% for both observables and in every village.

**Table C: difference between model simulations and target values (median of accepted runs)**

| **Observable** | **% of all NCC cases that have extra-parenchymal lesions** | **%of all clinical cases with ICH or hydrocephalus that only have parenchymal lesions** |
| --- | --- | --- |
| Observed/estimated value | 3.0% | 8.6% |
| Simulated, village 515 | 3.1% | 8.6% |
| Simulated, village 566 | 3.0% | 8.7% |
| Simulated, village 567 | 3.0% | 8.2% |
| *Average 3 villages* | *3.0%* | *8.4%* |
